# Stabilizing the Localized Surface Plasmon Resonance (LSPR) of Citrate-Synthesized Metal Nanoparticles in Organic Solvents

**DOI:** 10.3390/ma18225246

**Published:** 2025-11-20

**Authors:** Jacob P. Magdon, Matthew J. Jasienski, Madison R. Waltz, Gabrielle A. Grzymski, Calvin Chen, Arion M. Solomon, Minh Dang Nguyen, Jong Moon Lee, John C. Deàk, T. Randall Lee, Riddhiman Medhi

**Affiliations:** 1 Department of Chemistry, University of Scranton, 800 Linden Street, Scranton, PA 18510, USA; jacobmagdon@gmail.com (J.P.M.); matthew.jasienski@scranton.edu (M.J.J.); calvin.chen@scranton.edu (C.C.); arion.solomon@scranton.edu (A.M.S.);; 2 Department of Chemistry and the Texas Center for Superconductivity, University of Houston, 4800 Calhoun Road, Houston, TX 77204-5003, USA; dangminh27498@gmail.com (M.D.N.); trlee@uh.edu (T.R.L.)

**Keywords:** localized surface plasmon resonance, silver nanoparticles, gold nanoparticles, gold–silver nanoshells, phase-transfer, colloidal stability

## Abstract

Gold–silver nanoshells (GS-NSs) are hollow spherical nanoparticles with an alloyed Ag-Au shell. GS-NSs exhibit a tunable localized surface plasmon resonance (LSPR) in the visible to near-IR wavelengths as a function of composition and shell thickness and offer greater stability across pH ranges compared to other metal nanoparticles. These properties make GS-NSs promising materials for diagnostics, photothermal therapy, and photocatalysis. However, current research has explored GS-NSs only in aqueous systems, since they immediately aggregate in other solvents, limiting their utility. This paper provides an in-depth study of the choice and effect of non-thiol ligands on the stability and phase-transfer of GS-NSs from aqueous to non-aqueous solvents, such as ethylene glycol, tetrahydrofuran, dichloromethane, and toluene. Ligand exchange for functionalization of GS-NSs was performed with Triton X-100 (TX100), sodium stearate (NaSt), polyvinylpyrrolidone (PVP), and hydroxypropyl cellulose (HPC), prior to phase-transfer. The nanoparticles were phase-transferred to the non-aqueous solvents, and the stability of the colloids in the various solvents before and after functionalization was recorded with UV–visible spectroscopy, dynamic light scattering (DLS), zeta potential (ζ), scanning electron microscopy (SEM), and transmission electron microscopy (TEM). The study was also extended to include silver nanoparticles (AgNPs) and gold nanoparticles (AuNPs) to evaluate broad-range applicability. Among the ligands studied, HPC functionalization demonstrated the widest range of phase-transfer stability across 21 days for all three particle systems studied. UV–vis spectroscopy demonstrated sustained LSPR integrity after HPC functionalization in EG, THF, and DCM. SEM, TEM, and hydrodynamic size measurements by DLS further confirmed no aggregation in EG, THF, and DCM but suggested possible twinning or clustering in the solution. Overall, this work successfully identified non-toxic alternatives to expand the LSPR stability of citrate-synthesized metal nanoparticles in organic solvents.

## 1. Introduction

Nanoparticles have been studied for over a century, with the first paper published in 1857 by Michael Faraday [1]. Scientists are intrigued by nanoparticles, as the properties exhibited differ from the bulk material by way of localized surface plasmon resonance (LSPR), an oscillation of the electron cloud on the surface of nanoparticles in resonance with incident light [2,3]. This phenomenon can often be observed in colloidal plasmonic metallic nanoparticles, such as silver nanoparticles (AgNPs) and gold nanoparticles (AuNPs), which can absorb wavelengths between 400 and 1000 nm, depending on their type, size, and shape [2,4]. Localized surface plasmon resonance in a metal particle has been used for optics [5,6,7], sensing [8,9], catalysis [10,11,12,13,14,15], biomedical diagnostics and therapeutics [3,16,17,18,19,20,21], semiconductors [22,23], solar cells [4], and hydrogen production [24,25]. Nanoparticles are formed from various methods, such as citrate stabilization, often in aqueous solution, which forms 1–100 nm-sized colloidal nanoparticles held together by metallic bonding and colloidally separated via electrostatic or steric repulsions [12,22,26,27,28,29,30,31,32,33]. While researchers have made significant progress in recent times with the synthesis of colloidal metal nanoparticles in various shapes, sizes, and compositions, a challenge for these colloidal nanoparticles is the phase-transfer of these nanoparticles from typically aqueous systems to less polar solvents and vice versa [34] in order to expand the scope of these plasmonic nanoparticles. Phase-transfer is the method by which a substance dissolved or dispersed in one solvent is successfully transferred to another solvent while retaining comparable solubility or stability. Successful phase-transfer to a broader range of solvents expands the chemical possibilities for further synthetic modifications and catalysis [35,36]. For example, successful phase-transfer to organic solvents would allow us to leverage established non-aqueous methods like controlled hydrolysis and oleic acid/oleylamine-mediated synthesis of metal oxide nanocrystals and quantum dots for core–shell/core–seed nanocomposites or controlled radical polymerization methods for the synthesis of polymer-grafted nanoparticles (PGNs) [36,37,38,39,40].

Particle stabilization can be achieved through various interactions, including electrostatic, dipole–dipole, steric, and hydrogen bond interactions between ligands and solvents. Colloidal plasmonic nanoparticles synthesized via the widely used citrate method are stabilized in aqueous solution primarily by electrostatic interactions between surface citrate ions, which are disrupted in nonpolar solvents, leading to immediate and irreversible aggregation. Aggregation of colloidal plasmonic nanoparticles results in a complete loss of their optical LSPR properties, which has limited their exploration in non-aqueous systems [41]. This study aims to modify the surface interactions of these nanoparticles via ligand exchange [29,30] and potentially discover alternate agents to enable successful phase-transfer to non-aqueous solvents. An example of these methods, the transfer of gold nanoparticles from an aqueous phase to an organic phase, was reported by Soliwoda et al. [22] using steric interactions of an alkyl chain length of secondary amines from water to toluene for use in semiconductors and used the Brust–Schiffrin method of capping to transfer [31]. The findings showed that long alkyl thiol chains imparted hydrophobicity to gold nanoparticles, driving their transfer to the organic phase.

Recently, *N*-heterocyclic carbenes (NHCs) have shown great promise in phase-transfer to biological media, including buffers, cell culture media, and human serum, without thiols [18,42]. Similarly, some other articles have also been published that focus on ligands that do not include thiols, amines, or non-ionic liquids for green chemistry [15]. Thiols and amines have adverse effects on the planet, so finding stable ligands that are inert to the ecosystem is important [43]. This can be true for using nanoparticles in humans as well, as thiols and amines can have negative effects on the body [11,42]. In this research, we examine ligands commonly used in industry and pharmaceuticals that are not known to have adverse effects on the body or the planet. We recognize that this green methodology in functionalization and phase-transfers of nanoparticles is important for long-term sustainability.

The colloidal particles studied in this paper are GS-NSs, which are hollow nanoshells made from Au/Ag alloy, synthesized from citrate-stabilized AgNPs. Citrate-stabilized nanoparticles are widely used in industry and research, so phase-transfer strategies for citrate-stabilized nanoparticles can be directly applied by scientists who synthesize their plasmonic nanoparticles using the citrate method [44]. Also, while some reports have been published on the phase-transfer of metal nanoparticles such as gold, silver, platinum, and copper, researchers have yet to investigate phase-transfers for nanoshells such as gold–silver nanoshells (GS-NSs) presented here [12,15,20,22,29,30,32,35,45,46,47,48,49,50,51,52,53,54]. Nanoshells have unique LSPR that can be modified depending on the inner and outer shell thickness, composition, shape, and diameter [55]. This phenomenon of plasmon resonance leads to countless opportunities in regard to nanophotonics, with the ability to design nanoshells with a specific wavelength of extinction as desired [5,56,57]. GS-NSs are, therefore, promising materials for diagnostics, photothermal therapy, and photocatalysis [25,57,58]. While GS-NSs have shown greater stability than AgNPs and AuNPs in aqueous solutions across different pH ranges, attempts to transfer GS-NSs to non-aqueous systems still encounter the same challenges as particles synthesized using the standard citrate method, severely limiting their exploration in non-aqueous systems.

**Figure 1:**
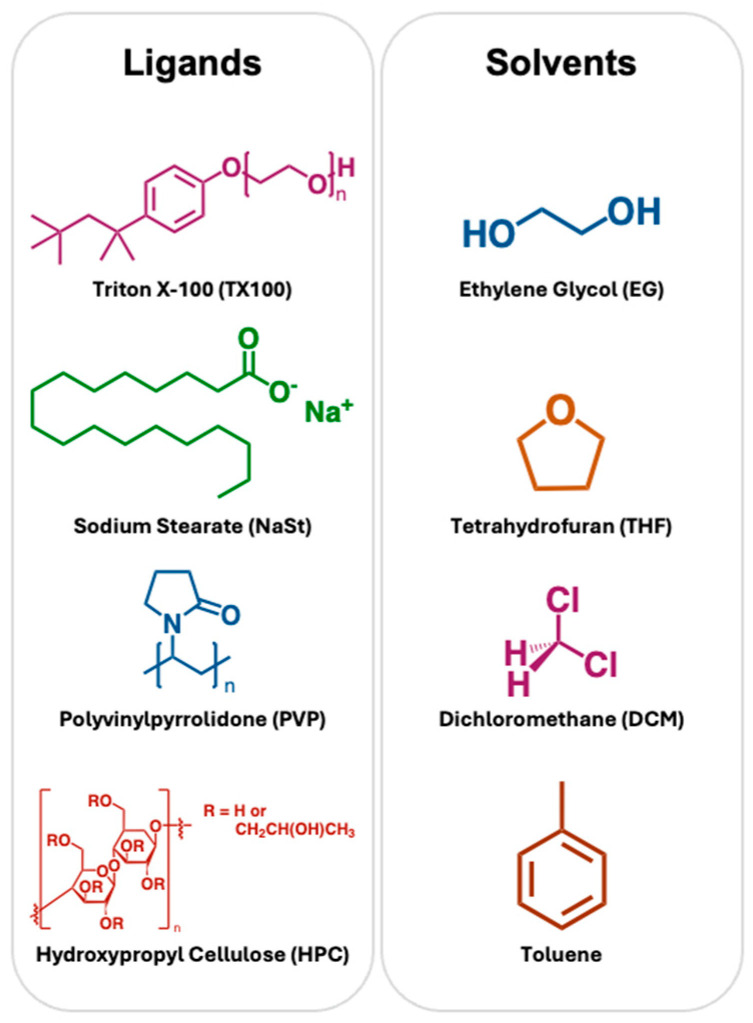
Ligands used in phase-transfer: Triton X-100 (TX100, violet), sodium stearate (NaSt, green), polyvinylpyrrolidone (PVP, blue), and hydroxypropyl cellulose (HPC, red). Solvents tested for phase-transfer: ethylene glycol (EG, blue), tetrahydrofuran (THF, orange), dichloromethane (DCM, purple), and toluene (brown). These colors match up to later graphs.

Specifically, this study investigates the ligand exchange of citrate-stabilized aqueous gold–silver nanoshells (GS-NSs) with four ligands: hydroxypropyl cellulose (HPC), polyvinylpyrrolidone (PVP), sodium stearate (NaSt), and Triton-X 100 (TX100) and their subsequent phase-transfer into the solvents ethylene glycol (EG), dichloromethane (DCM), tetrahydrofuran (THF), and toluene (see Figure 1). These ligands were chosen as alternatives to traditional amine- and thiol-based agents for improved sustainability. The GS-NSs underwent a thorough study on the ligand exchange, phase-transfer, and LSPR stability using dynamic light scattering (DLS), scanning and transmission electron microscopy (SEM/TEM), zeta potential (ζ), and UV–visible spectroscopy to understand the fundamental interactions of these functionalization and phase-transfer processes. The main goal of this work is to investigate the phase-transfer of nanoshells synthesized through the citrate method as they undergo ligand exchange in order to find the most versatile ligand, so that other researchers can utilize nanoshells in non-aqueous chemistry without diverging from their established citrate-based synthetic methods that are already used.

## 2. Experimental Methods

Materials and characterization methods, as well as the synthetic procedures and parameters used to synthesize the silver nanoparticles (AgNPs), gold–silver nanoshells (GS-NSs), and gold nanoparticles (AuNPs), are detailed in the Supplementary Materials Section S1.

### 2.1. Functionalization

To ensure consistency and reproducibility, we synthesized a master batch of AgNPs, AuNPs, and GS-NSs that were used for the entire study. For ligand functionalization of GS-NSs and AgNPs, 0.0200 g of ligand was added to a 10 mL portion of GS-NSs or AgNPs and sonicated for 90 min, followed by centrifugation to remove water and redispersed in 10 mL of absolute ethanol. Subsequently, an additional 0.0200 g of the ligand was added to the vial. The vial was then mixed and sonicated for 90 min. The ligands added were HPC, PVP, sodium stearate (NaSt), and Triton-X 100 (TX100). For ligand functionalization of AuNPs, 0.0400 g of ligand was added to a 20 mL portion of AuNPs and sonicated for 15 min. Nanoparticles remained dispersed in the water. Ligands added were HPC, PVP, and TX100. We did not use NaSt for the AuNP study, since the GS-NS and AgNP results showed aggregation with NaSt in most of the solvents.

### 2.2. Phase-Transfer

**Scheme 1:**
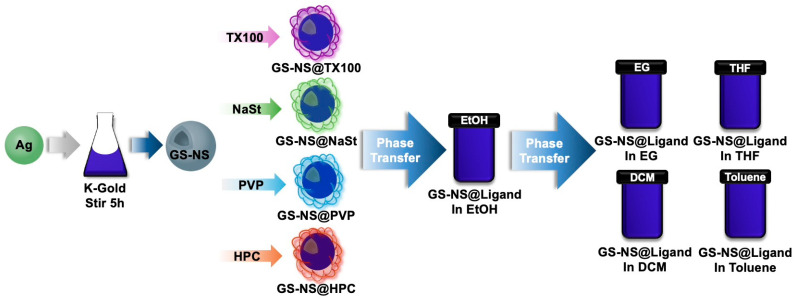
Synthesis, functionalization, and phase-transfer of GS-NSs.

The phase-transfers of GS-NSs and AgNPs were performed by centrifugation to remove ethanol, add the solvent to the GS-NS-ligand, and sonication for 15 min. This step is where aggregation tends to begin, so observations and swift characterizations are vital. These phase-transferred particles were stored in the dark at room temperature. An overview of the entire process can be seen in Scheme 1. For any anomalous observations, samples were reprepared and measurements repeated to ensure reproducibility.

Phase-transfer of AuNPs was performed by the addition of solvent to the AuNP@Ligand (where Ligand = TX100/NaSt/PVP/HPC) aqueous solution and thoroughly shaking the vial directly (no centrifugation and redispersion were employed for AuNPs). The vials were then stored in the dark at room temperature. For the miscible solvents, the UV–vis spectra were recorded for the whole solution, while, for the phase-separated solvents (DCM and toluene), only the organic layer was extracted for the UV–vis spectra.

## 3. Results and Discussion

### 3.1. Synthesis and Morphology

The AgNPs synthesized followed the reported procedure [4] originally adopted from Li et al. [59]. The UV–vis spectra showed a localized surface plasmon resonance peak with a λ_max_ between 405 and 410 nm (see Figure S1). The AgNPs were combined into a master batch for this study to eliminate any differences from batch synthesis. The UV–vis of the combined batch in Figure S2 showed a λ_max_ at 405 nm, which is consistent with the synthetic techniques.

The GS-NSs were synthesized from the combined AgNP master batch via a galvanic replacement method reported previously [4]. The dielectric core of the AgNPs is etched out as a thin layer of gold added to the silver in a galvanic replacement process, yielding a hollow alloyed shell [41]. To change the LSPR λ_max_, the K-gold amount (K_2_CO_3_ + HAuCl_4_) and time can be controlled as reported previously [4]. GS-NSs in this study were synthesized from the master AgNPs in batches and later combined, and the UV–vis spectra (diluted 5x) of the individual batches and the combined batch for GS-NSs are shown in Figures S3 and S4. Table 1 below lists the key properties of the GS-NSs before and after functionalization and phase-transfer, such as the wavelength of maximum extinction (λ_max_), the zeta potential (ζ), which is a measure of the surface charge on the particles, the hydrodynamic diameter obtained from DLS measurements, and the particle size measured from SEM images. The combined batch of GS-NSs has a hydrodynamic diameter of 74.11 nm and a λ_max_ at 750 nm.

### 3.2. Functionalization and Time-Resolved Phase-Transfer of GS-NSs

#### 3.2.1. Ligand Exchange and Phase-Transfer Protocol for GS-NSs

Our primary study was centered around the ligand exchange and phase-transfer of GS-NSs, and the ligands explored in this study are illustrated in Figure 1. The ligands were chosen based on initial stability in water for functionalization, and the subsequent possibility of polar/nonpolar interactions, hydrogen bonding, and steric repulsion within the various organic solvents. TX100 and NaSt are common surfactants used for stabilizing nanoparticles of varying shapes, and TX100 has been seen to be particularly effective in stabilizing notoriously unstable structures like nanostars [60]. There is a considerable hydrocarbon component in NaSt and TX100, which could help disperse nanoparticles in certain organic solvents. NaSt specifically was thought to bind to the outer surface of the GS-NSs through the carboxyl, while the long alkyl chain imparts stability in the organic solvents. TX100 is a versatile surfactant because of the hydrophilic polyethylene oxide chain and has a hydrophobic aromatic hydrocarbon lipophilic group. PVP and HPC were both chosen for their established solubility in water for functionalization, given by the presence of polar carbonyl groups and hydroxyl groups present in the molecules, respectively, and a hypothesis that a phase-transfer into other less polar solvents was possible due to the overall hydrophobic backbone and H-bonding groups present.

The hydrodynamic diameter, SEM images, and the zeta potential of the nanoshells were studied before and after functionalization in water (Figures S5 and S6). After functionalization, the SEM images did not indicate any changes on a particulate level; however, the changes in DLS diameter (see Table 1) can be an indicator that the nanoparticles have undergone some changes in stability, and a slightly larger hydrodynamic diameter may be a resultant of either aggregation in nanoparticles in groups of twos or threes.

After the addition of the HPC ligand, the zeta potential changed to −28.5 mV. As HPC is not a charged molecule, a neutralized zeta potential was expected with complete ligand exchange and surface coverage; however, only a partial neutralization of the negative zeta potential suggests some citrate ions potentially still occupying part of the surface. DLS data also increased from 74.11 nm to 96.17 nm. PVP zeta potential only slightly changed when in water from −42.0 mV to −42.8 mV, and the hydrodynamic diameter increased from 74.11 nm to 76.09 nm. These changes are not significant enough to show that a ligand exchange occurred. However, when the GS-NS@PVP was phase-transferred from water to ethanol, a significant shift in results followed. The zeta potential changed from −42.8 mV to −1.21 mV, which was originally expected due to the uncharged nature of PVP. The DLS data also had an increase from 76.09 nm to 144.9 nm. A couple of possibilities are theorized. PVP can take an extended amount of time to exchange ligands with the citrate ions due to steric hindrance naturally found with large polymer chains, resulting in a possibility that a complete ligand exchange never occurred initially in water, and when the GS-NS@PVP was phase-transferred from the water to ethanol, only the PVP-stabilized GS-NSs would have survived, while the citrate-functionalized particles crashed out, thus more accurately reflecting the expected zeta potential for PVP-coated particles, given that a concentration decrease was also observed after dispersing in ethanol.

For the GS-NS@NaSt, the zeta potential changed to −51.4 mV after ligand exchange with NaSt. NaSt is a charged system, so a large negative zeta potential was expected. The hydrodynamic diameter increased from 74.11 nm to 87.06 nm. From these data, a successful ligand exchange with NaSt can be concluded. GS-NS@TX100 was interesting, showing a zeta potential change from −42.0 mV to −31.3 mV, with a marginal increase in DLS diameter from 74.11 nm to 76.87 nm. A change in zeta potential confirmed a ligand exchange occurred and the hydrodynamic diameter remained stagnant, which could mean that either GS-NS@TX100 and GS-NS with citrate form a similar colloidal size in water or the possibility of an incomplete ligand exchange. Figure S6 shows the SEM images of the nanoshell with the ligands in water. All the particles remain discrete in water after functionalization.

In most prior works with phase-transfer studies [35], the organic solvent is only added to the nanoparticles in ethanol or water and shaken, leaving remnant water or ethanol, resulting in a miscible or immiscible solvent system, thus making it difficult to determine whether the organic solvent is interacting directly with the nanoparticles or via a solvation medium. The method utilized here eliminated water and ethanol via centrifugation prior to dispersion in the organic solvent, so the interactions observed are strictly between the functionalized particles and the target organic solvent. The target solvents shown in Figure 1 were chosen for their wide range of applications and differences in polarity to each other. DCM is a widely used organic solvent that has some polarity. The polar ligands, HPC and PVP, are expected to interact favorably, enabling a phase-transfer. While DCM is a tetrahedral molecule, THF is a heterocyclic molecule but also possesses an overall dipole. Ethylene glycol is a more viscous solvent with O-H dipoles, similar to ethanol. Toluene is largely nonpolar but has been known to transfer AuNPs in the past [12,22], and GS-NSs exhibit more Au character on the surface.

#### 3.2.2. Phase-Transfer of GS-NS@TritonX-100

GS-NS@TX100 was stable when phase-transferred to EG over the entire 21-day period. There was a decrease in nanoparticle concentration, resulting from centrifugal processes for removing water and ethanol from samples. TX100 has been previously researched on nanoparticles to form a reverse micelle. This reverse micelle GS-NS@TX100 has a polar ether backbone available for interactions at the ligand–solvent interface [61]. The intermolecular hydrogen bonding within EG leads to the high viscosity of EG, which can also help suspend particles and restrict the nanoshells from aggregation. The time-resolved UV–vis extinction spectra in Figure S7 show little change compared to the initial GS-NSs in water. The inset graphs in Figure S7 show no significant change in the λ_max_ over time. GS-NS@TX100 phase-transferred to THF successfully but with more aggregation present. A significant λ_max_ shift did not occur until day 21, when a blue shift occurred, but it was not significant, as the original peak remained. The polar ether chain formed dipole–dipole interactions with THF, and the steric hindrance was sufficient to form stable colloids.

GS-NS@TX100 initially phase-transferred to DCM with the λ_max_ increasing from 717 nm to 794 nm (see Figure S7). The UV–vis spectra started showing aggregation from d = 0, which was a sign that GS-NS@TX100 was not going to survive over time in DCM. The existing zeta potential of −31.3 mV after ligand exchange, although TX100 is a nonionic ligand, indicates TX100 is probably also not large enough to form a thorough coating of the entire nanoshell. Additionally, the steric size of the backbone is too small to overcome the weak attraction, and the nanoshells aggregate over time.

A phase-transfer of GS-NS@TX100 into toluene had anomalous results. Complete aggregation started at d = 0 and stayed until d = 21 when the GS-NS@TX100 unexpectedly re-exhibited an LSPR signal in toluene, as shown in Figure S7. The colloids were also confirmed to be much larger by DLS, as the hydrodynamic diameter was 648.4 nm. This phenomenon has never been documented, as of our findings where nanoparticles were aggregated and became colloidal in a solvent after a period of time since aggregation of metallic colloids were generally irreversible. The colloids were confirmed by SEM imaging as nanoshells with small particles and larger aggregates of differing morphology in Figure S8, and the results were replicated in additional trials.

**Figure 2:**
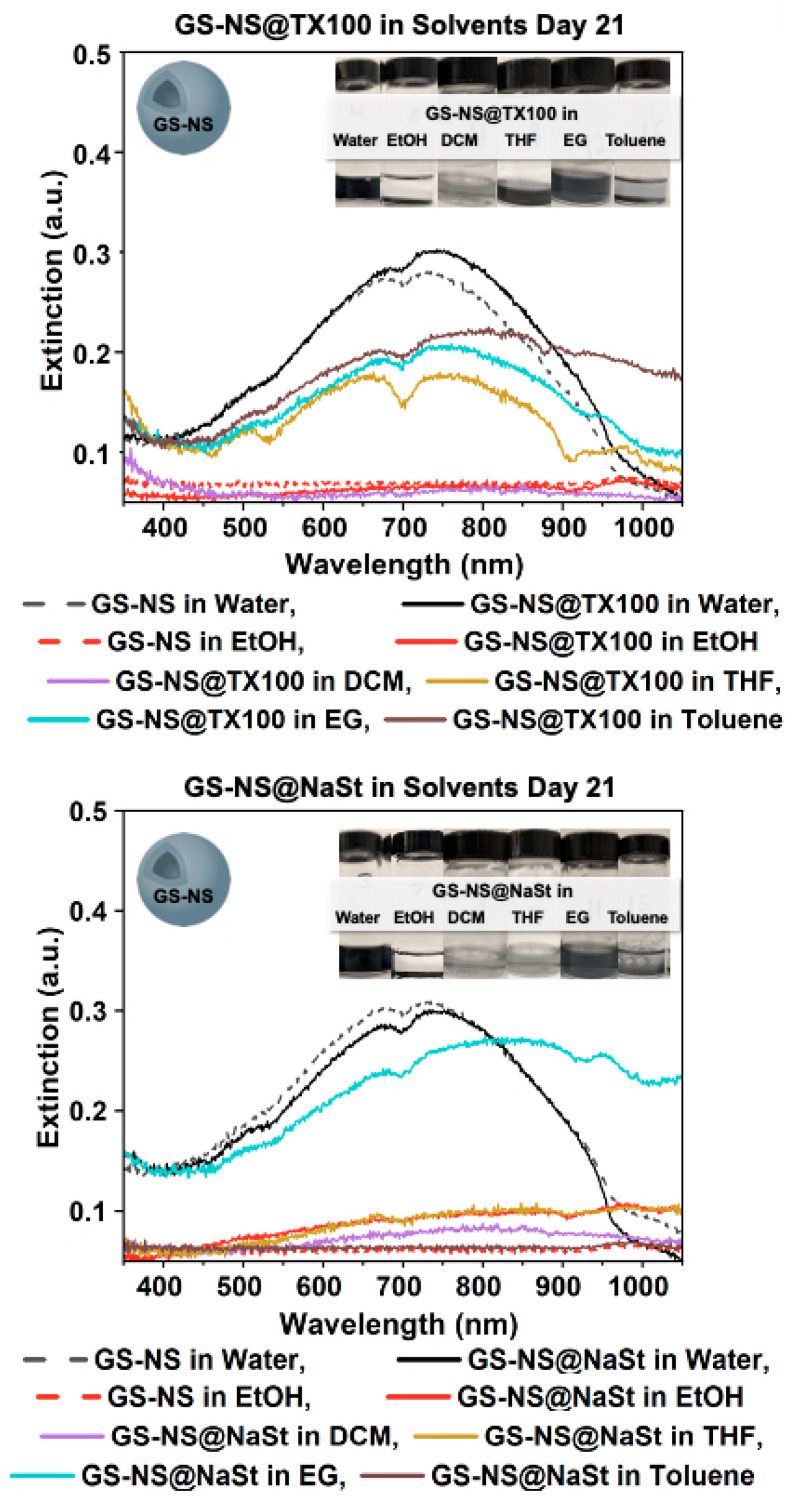
UV–vis spectra for GS-NS@TritonX-100 and GS-NS@NaSt after day 21 in water, EtOH, EG, THF, DCM, and toluene. GS-NS@TX100 were stable in EG, THF, and toluene; GS-NS@NaSt were only stable in EG.

The UV–vis spectrum of GS-NS@TX100 was plotted in all solvents in Figure 2 to complete a parallel comparison after d = 21. GS-NS@TX100 does not survive in ethanol or DCM. Both solvents have polar groups, showing hydrogen bonds are not enough for phase-transfer. An ethanol-like molecule (1-butonal) has previously been shown to exchange ligands with nanoparticles when TX100 is attached [62]. TX100 does not completely envelop a nanoparticle, but instead, the hydrophilic polyethylene oxide chain is linear and pointed away from the nanoparticle with a small steric size. Over time, nanoshells aggregate, as the steric hindrance is not enough to keep the nanoshells apart. A similar conclusion is possible for GS-NS@TX100 in DCM.

GS-NS@TX100 phase-transferred into THF and EG and survived for the 21-day period of the experiment. Good stability was found in EG, as the spectrum retained the same shape as water, showing little change in particle morphology. GS-NS@TX100 into toluene is a unique case where an unknown interaction is observed between the ligand and solvent on d = 21, only redispersing the particles, but this is most likely an anomaly and cannot be taken as an indication of long-term stability.

#### 3.2.3. Phase-Transfer of GS-NS@NaSt

NaSt has a carbon chain available for the surface–solvent interface, while the carboxyl is bonded with the nanoshells. GS-NS@NaSt partially aggregated when phase-transferred to EG. A peak remains at 750 nm with a redshift, which signifies small aggregates of nanoshells that are seen in Figure 2, which overall remain colloidal due to a slight hydrophobicity of the NaSt and viscosity of EG preventing nanoshells from aggregation. GS-NS@NaSt in THF and DCM only phase-transferred for d = 0 before aggregation, as shown in the UV–vis spectrum of Figure S9. This phase-transfer was not stable, as the NaSt attaches to GS-NSs at the carboxyl group and has an alkyl chain that has no polar groups to stabilize phase-transfer in THF or DCM long-term or to sterically hinder the nanoshells from aggregating [63]. GS-NS@NaSt did not phase-transfer into toluene at all. The comparison of NaSt in all solvents at d = 21 shows that it only was stable long-term in EG, perhaps pertaining to EG’s viscosity when stabilizing the NP colloids by suspension in the solvent. NaSt was not able to develop a hydrogen bond due to the lack of polar groups available and was too small to sterically stabilize in EtOH, DCM, THF, and toluene.

#### 3.2.4. Phase-Transfer of GS-NS@PVP

**Figure 3:**
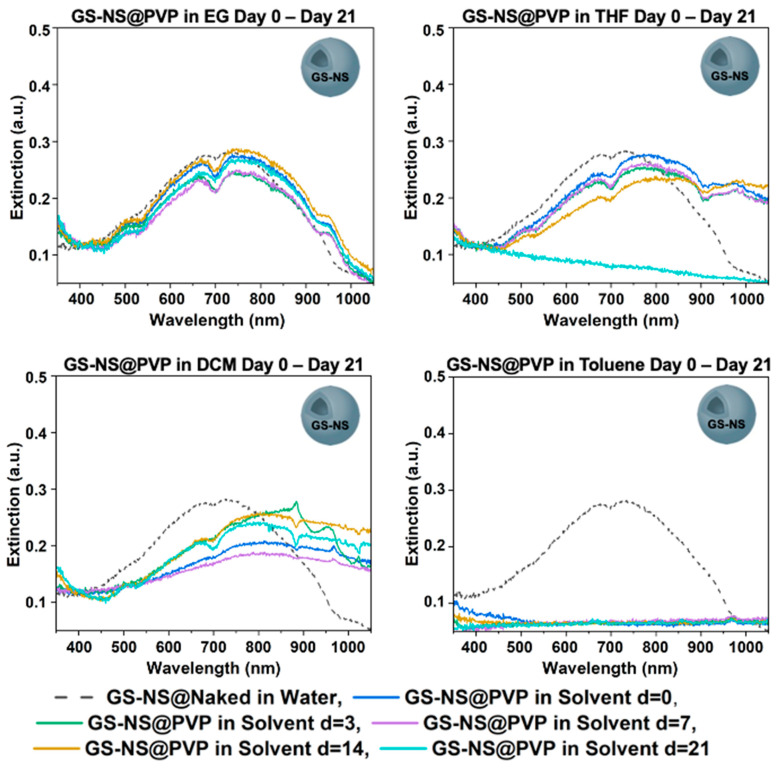
UV–vis spectra for GS-NS@PVP after days 0, 3, 7, 14, and 21 in different solvents.

GS-NS@PVP was shown to have an almost full ligand exchange with citrate, with a zeta potential of −1.21 mV after ligand exchange. The structure of PVP has both carbonyl and nitrogen that can bond to the nanoshells [64]. As PVP is a polymer, a complete micelle-like coating surrounds the particle, as determined from the zeta potential data. GS-NS@PVP phase-transferred into EG with high colloidal stability, evident by a small change in the UV–vis spectrum in Figure 3. EG can greatly form a hydrogen bond with the oxygen from the ketone found in PVP, which leads to this great stability coupled with EG’s internal viscosity. SEM images taken of GS-NS@PVP in EG at day 21, presented in Figure S10, confirmed that the nanoshells maintained their morphology.

GS-NS@PVP was colloidal in THF until day 21, but after d = 14, the colloid aggregated. The time spectrum of the phase-transfer showed aggregation slowly occurring over 14 days, indicating that the interactions were not strong enough to sustain colloidal stability long-term in THF. The exact reason for this slow aggregation needs further investigation, but limited hydrogen bonding could be a plausible reason, suggesting the interactions were not strong enough to sustain a colloidal solution long-term.

When transferred to DCM, GS-NS@PVP had an increase in larger particles but maintained colloidal stability throughout the 21 days. A large red shift occurred from 727 to 812 immediately on the UV–vis of Figure 3, which remained around that range. The larger particles were confirmed by the SEM image of the nanoshells in Figure S10. DCM contains chlorines, which are electron-withdrawing, pulling the electrons towards the chlorine, leaving the hydrogens with a polar partial positive charge that the polar oxygen on PVP can establish a weak interaction with. PVP is also a polymer with a large steric size to stabilize nanoshell colloids for a long period of time. GS-NS@PVP did not phase-transfer into toluene, as evident in the UV–vis spectra of Figure 3. Similar to the previous GS-NS@NaSt, toluene is nonpolar and does not have any polar interactions with the PVP ligand. These results were expected and were useful in understanding the polar effects on solvent–ligand interactions.

**Figure 4:**
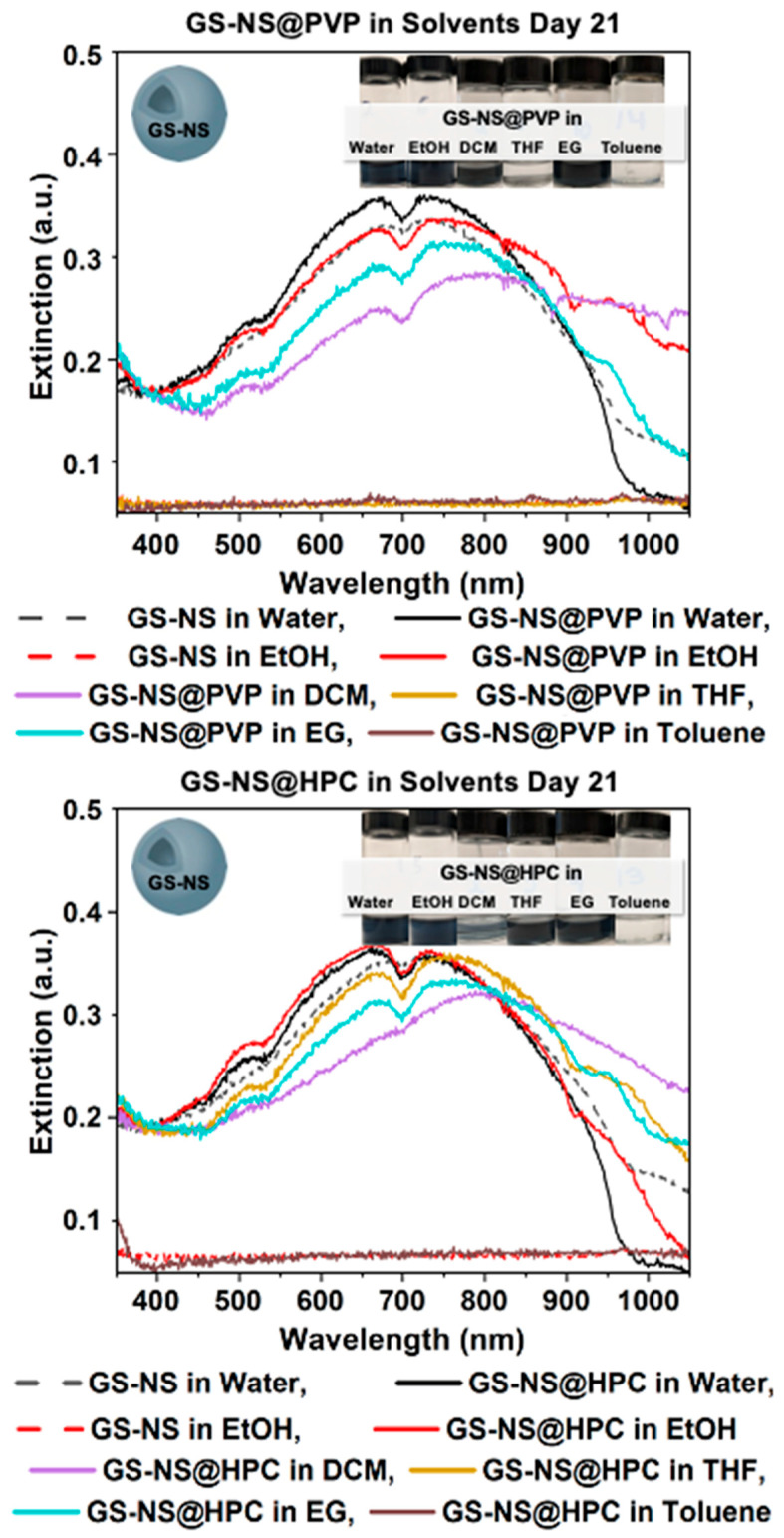
UV–vis spectra for GS-NS@PVP and GS-NS@HPC after day 21 in water, EtOH, EG, THF, DCM, and toluene. GS-NS@PVP was stable in EtOH, EG, and DCM; GS-NS@HPC was stable in all solvents except toluene.

The following spectra in Figure 4 are GS-NS@PVP in each of the solvents graphed at day 21 to show which solvent was most effective at phase-transfer long-term. For GS-NS@PVP into EG, a greater stability was found than in ethanol and DCM. This stability makes sense, as EG has greater polarity and viscosity, which stabilizes the nanoshells in solution. When phase-transferred to DCM, an increase in larger particles was present, as a λ_max_ shift of 61 nm was found, but the colloid was stable for 21 days. GS-NS@PVP did not survive 21 days when phase-transferred to THF, while they immediately aggregated in toluene.

#### 3.2.5. Phase-Transfer of GS-NS@HPC

**Figure 5:**
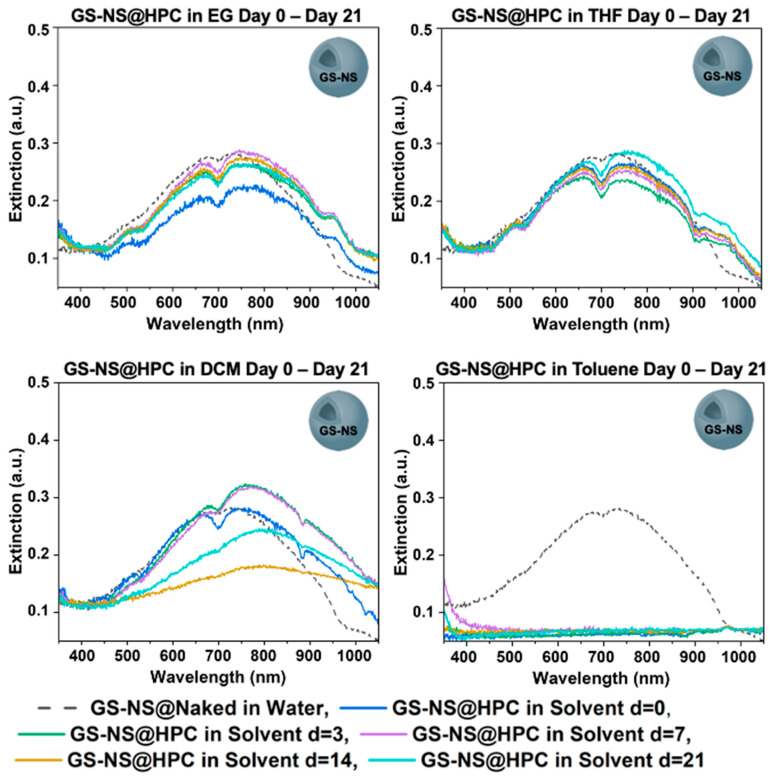
UV–vis spectra for GS-NS@HPC after days 0, 3, 7, 14, and 21 in different solvents.

HPC is a polymer with many polar groups available for hydrogen bonding and a cyclic structure based on cellulose with hydroxyl side groups. HPC was hypothesized to be our best ligand at phase-transfer due to the polar groups and large steric size. GS-NS@HPC underwent a partial ligand exchange, according to the zeta potentials dropping to −21.7 mV after ligand exchange. GS-NS@HPC in EG was stable due to the viscosity of EG and the number of polar groups available in HPC and EG to form hydrogen bonds. A slight red shift of under 40 nm from 727 nm to 760 nm occurred in the UV–vis spectra of Figure 5. A TEM image was taken of the particles after phase-transfer and is shown in Figure S11. In the TEM, a corona of the ligand can be seen, and the morphology remained unchanged after phase-transfer. GS-NS@HPC in THF showed stability based on the UV–vis and TEM (see Figure 5, Figures S11 and S12), with a very small red shift of the λ_max_. TEM imaging showed the particles with a corona of ligand visible, and they were monodispersed and spherical. The reasoning is similar to GS-NS@HPC in EG, as the polar THF can have dipole–dipole interactions with HPC’s polar hydroxyl groups. Previous studies of water contact angles on surfaces coated with HPC (WCA = 57°) and PVP (WCA = 48°) have shown the greater hydrophility of HPC stemming from its polar hydroxyl groups, supporting this hypothesis [65]. The steric size of HPC is also large, further enhancing the long-term stability of the colloids.

GS-NS@HPC phase-transferred to DCM and remained colloidal, but more small aggregates were present. The UV–vis spectrum in Figure 5 shows that the λ_max_ red-shifted 61 nm after 21 days, but the LSPR broadening was significant in the 750–800 nm region, indicating some aggregation had occurred. This was confirmed by the DLS data as the hydrodynamic size increased to 221.7 nm. The interactions present are partial hydrogen bonds between HPC and DCM, given that the hydrogens on DCM are partially positive from the electron-withdrawing chlorines nearby. These partial hydrogen bonds would be weaker than the hydrogen bonds between GS-NS@HPC with EG or THF. The steric size of the HPC is an additional factor that kept the colloids from aggregating. Morphology remained unchanged, as seen in the SEM image of Figure S13. Meanwhile, GS-NS@HPC did not phase-transfer to toluene at all, as can be seen in the UV–vis of Figure 5. Toluene is nonpolar, while HPC has polar groups and is amphiphilic at best. The nonpolar component is not strong enough to sustain the colloids, and steric repulsions between the functionalized particles are not sufficient to make up for it.

**Figure 6:**
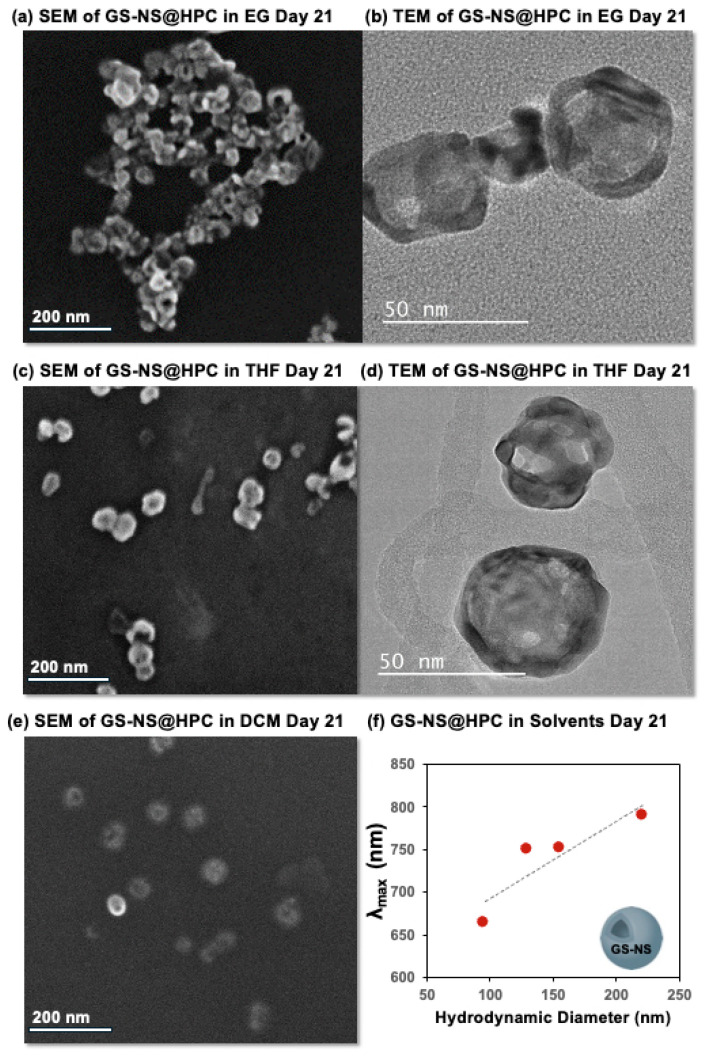
(**a**–**e**) SEM and TEM images of GS-NS@HPC after day 21 in EG, THF, and DCM. (**f**) Scatter plot (and trendline) for the hydrodynamic diameter vs. λ_max_ for GS-NS@HPC after day 21 in different solvents.

The UV–vis spectra in Figure 4 and the SEM and TEM images in Figure 6, offer a comparative analysis for GS-NS@HPC in each of the target solvents on day 21. When phase-transferred to EG and THF, a red shift is observed, and the spectra largely remain similar. DCM showed LSPR broadening in the 750–800 region brought on by the partial aggregation of particles, while complete aggregation was observed with toluene. The results can be rationalized by the more polar interactions present for GS-NS@HPC with THF and EG, while less polar interactions are present with DCM, and no polar interactions or hydrogen bonding are present with toluene. While Figure 6a–e show that the particles are mostly discrete in the deposited SEM and TEM samples from these solvents, the hydrodynamic diameters in Figure 6f clearly show a correlation with the LSPR shift and broadening in EG, THF, and DCM. This indicates, as described before, that, while the particles do not completely aggregate out, there is likely some partial aggregation in twos or threes in the dispersed colloidal state, especially in DCM.

### 3.3. Comparative Analysis of the Final Ligand Stability for GS-NSs

The previous section detailed how stable a ligand is in a solvent and provided a summary of the ligand in all the solvents at 21 days. The following section will compare GS-NS@HPC, GS-NS@PVP, GS-NS@NaSt, and GS-NS@TX100 in a solvent to determine which ligand is the best for long-term phase-transfer.

#### 3.3.1. Final Stability of GS-NS@Ligand in EG

**Figure 7:**
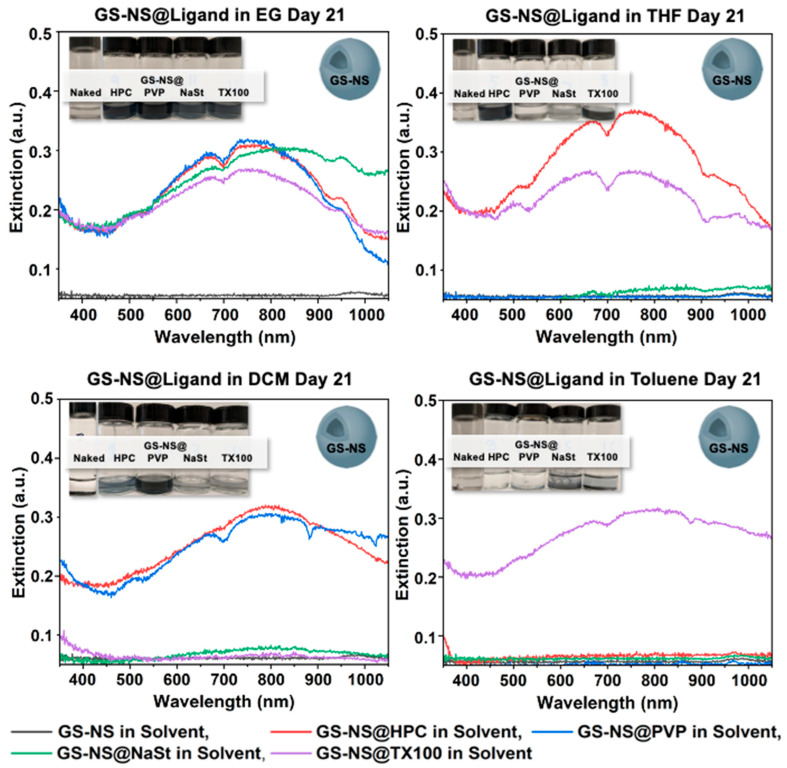
UV–vis spectra for GS-NS, GS-NS@TX100, GS-NS@NaSt, GS-NS@PVP, and GS-NS@HPC after day 21 in EG, THF, DCM, and toluene.

When GS-NS@HPC, GS-NS@PVP, GS-NS@NaSt, and GS-NS@TX100 were phase-transferred to EG, all functionalized nanoshells survived. The results also show that ligand exchange is necessary for a stable colloid in EG, as the GS-NSs stabilized by citrate do not phase-transfer to EG. GS-NS@HPC and GS-NS@PVP were able to phase-transfer well to EG, with no significant changes in the morphology or UV–vis spectra in Figure 7 and Figure S12. EG (μ = 6.7 × 10^30^ cm) is more polar than EtOH (μ = 5.7 × 10^30^ cm) and has a higher hydrogen bond capacity with two O-H groups. Both HPC and PVP are largely stabilized by polar interactions in these colloids. GS-NS@TX100 phase-transferred and maintained a similar morphology to GS-NS@HPC and GS-NS@PVP but at a lower concentration. This can help confirm that a partial ligand exchange took place between TX100 and citrate, as the citrate-stabilized nanoshells aggregate out of the colloid, leading to a lower concentration overall. GS-NS@NaSt did phase-transfer from ethanol to EG but with a morphological change in particles shown by the UV–vis spectra, LSPR broadening of Figure 7, and SEM image of Figure S12. This is explained by the nonpolar, small steric size of the alkyl chain, leading to limited steric hindrance to nanoshell aggregation. Figure S12 shows SEM and TEM images in EG for GS-NS@HPC, GS-NS@PVP, and GS-NS@TX100, showing that the morphology is best maintained by GS-NS@HPC; GS-NS@NaSt did not survive the SEM prep. GS-NS@HPC and GS-NS@TX100 had hydrodynamic diameters of 129.4 and 97.73 nm, respectively, while the GS-NS@PVP diameter was 29.64 nm, and the DLS data further concluded the presence of stable colloids. While the refractive index (R.I.) of the surrounding medium has been known to influence the LSPR signal of metal nanoparticles [4], and different solvents have different R.I., we believe this factor is largely outweighed by the inherent stability and aggregation of the colloids in this study. As seen from the vials in the insets of Figure 2, Figure 4 and Figure 7, the LSPR signals in this study are clearly a function of the aggregation factor (and size) of the particles in this case, also confirmed by all the SEM and TEM images, and any effect on the R.I. would be negligible here.

#### 3.3.2. Final Stability of GS-NS@Ligand in THF

GS-NS@HPC phase-transferred to THF (μ = 5.7 × 10^30^ cm) with a hydrodynamic diameter of 154.9 nm, and the morphology did not change. The hydrogen bonding and electrostatic attractions between the HPC polar hydroxyls and the oxygen in THF stabilized the ligand in the solvent, and the steric size of the HPC showed stabilization of the colloids, as steric hindrance kept the nanoshells apart. The UV–vis spectra shown in Figure 7 supported that only GS-NS@HPC phase-transferred to THF with minimal change and survived the prep for SEM, while GS-NS@TX100 did not; the SEM image of GS-NS@HPC in THF is shown in Figure S13 and has spherical nanoshells. GS-NS@TX100 showed a weaker LSPR signal in THF for day 21, indicating that the incomplete ligand-exchanged particles aggregated out of solution, while the more complete GS-NS@TX100 remained colloidal until day 21 but aggregated soon afterward. GS-NS@PVP and GS-NS@NaSt both aggregated in THF by day 21. PVP is a polymeric system and could be expected to be similar for most phase-transfers with HPC. However, PVP does not phase-transfer into THF, which would suggest that a separate interaction coming from the O-H bonds in HPC, which are not present in PVP, is important in THF.

#### 3.3.3. Final Stability of GS-NS@Ligand in DCM

In the phase-transfers that were successful for DCM (μ = 6.0 × 10^30^ cm), a red shift occurred, indicating some aggregation was present. GS-NS@HPC and GS-NS@PVP both successfully phase-transferred into DCM. Both ligands were able to phase-transfer due to the large steric size and high number of polar groups for dipole–dipole interactions with DCM. GS-NS@HPC was slightly more efficient due to more polar groups present and a larger steric size, as shown by the UV–vis spectra in Figure 7, with slightly less red shift than GS-NS@PV. SEM images have little aggregates of the phase-transferred particles, which can be seen in Figure S14. GS-NS@NaSt and GS-NS@TX100 did not phase-transfer. This is likely due to the lack of polar groups in the backbone and a smaller steric size.

#### 3.3.4. Final Stability of GS-NS@Ligand in Toluene

Toluene is the most nonpolar solvent in our study (μ = 1.3 × 10^30^ cm). GS-NS@TX100 was the only ligand to show an LSPR signal in toluene on day 21. However, the colloids were completely aggregated until day 21, when a colloid dispersed back into the solution, so this was perhaps an anomaly rather than a reliable trend. There was also an apparent increase in large particles, as shown by the increase in LSPR broadening in the UV–vis spectra of Figure 7. In Figure S15, the SEM image of the particles and the DLS diameter of 648.4 nm confirmed the aggregation. None of the other ligands survived in toluene. This can be attributed to the fact that all ligands have polar components, and toluene is severely nonpolar. There are no polar interactions or hydrogen bonding possibilities in toluene, and as the results suggest, steric repulsions in HPC/PVP alone are not sufficient to overcome this complete lack of ligand–solvent interactions.

### 3.4. Functionalization and Time-Resolved Phase-Transfers of AgNPs

#### 3.4.1. Ligand Exchange and Phase-Transfer Protocol for AgNPs

**Figure 8:**
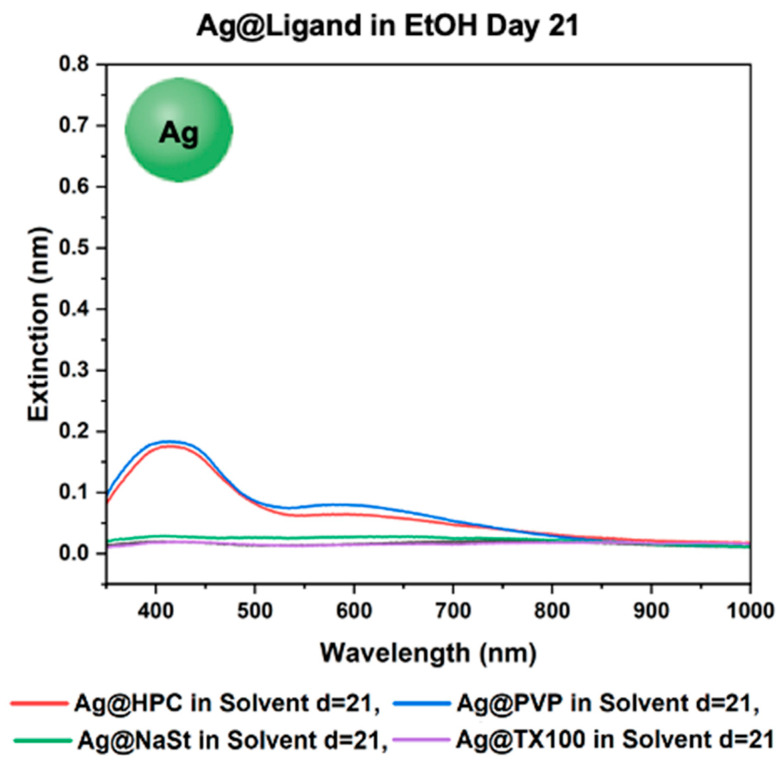
UV–vis spectra for Ag@Ligand in EtOH after day 21.

To observe if the ligand stability trends apply to other plasmonic metallic nanoparticle systems as well, we expanded the study to AgNPs. The ligand functionalization and phase-transfer protocols for the AgNPs were the same as that for GS-NSs. As seen in Figure 8, among the functionalized AgNPs, only Ag@PVP and Ag@HPC survived in EtOH after 21 days. Therefore, only the Ag@PVP and Ag@HPC systems were carried over for study in the four solvents.

#### 3.4.2. Phase-Transfer of Ag@PVP

**Figure 9:**
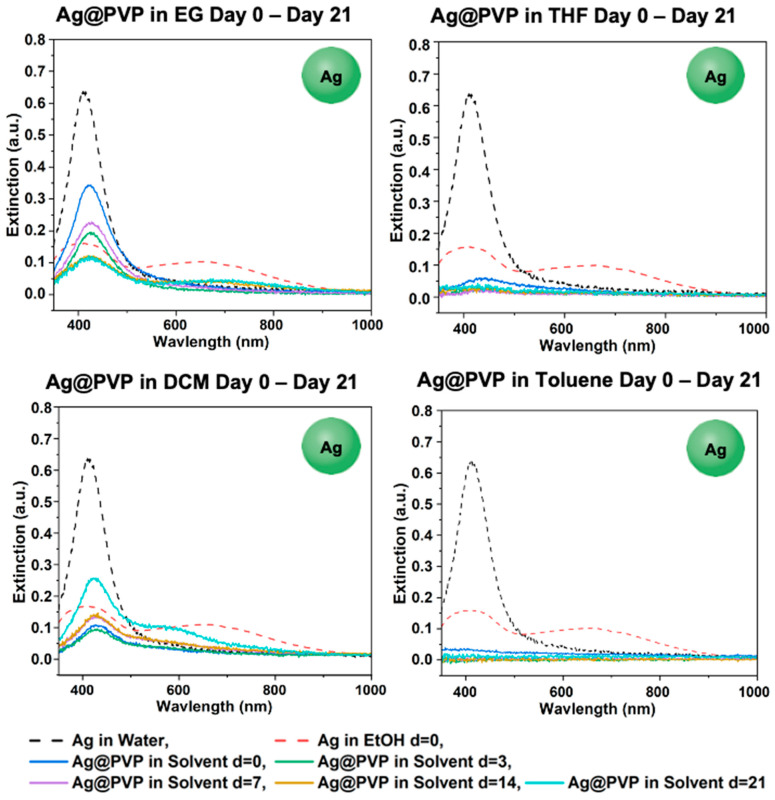
UV–vis spectra for Ag@PVP in EG, THF, DCM, and toluene after days 0, 3, 7, 14, and 21.

Figure 9 shows that Ag@PVP survived in EtOH, EG, and DCM throughout the 21-day period; however, the concentration in EG decreased throughout the 21-day period. Ag@PVP showed phase-transfer to THF on day 0 but aggregated between day 0 and day 3, while aggregation occurred nearly immediately in toluene. Comparing the state of Ag@PVP in each solvent on day 21, the nanoparticles survived phase-transfer into EtOH, EG, and DCM, with the highest concentration in DCM (Figure S16). However, the nanoparticles aggregated in THF and toluene, as mentioned before. This is relatively similar to the behavior of GS-NS@PVP in the same solvents, as both Ag@PVP and GS-NS@PVP survived phase-transfer to EtOH, EG, and DCM (see Figure 3, Figure 4, Figure 8 and Figure S16). Both Ag@PVP and GS-NS@PVP aggregated in toluene and THF. However, Ag@PVP in THF aggregated on day 3, while GS-NS@PVP in THF survived until day 21.

#### 3.4.3. Phase-Transfer of Ag@HPC

**Figure 10:**
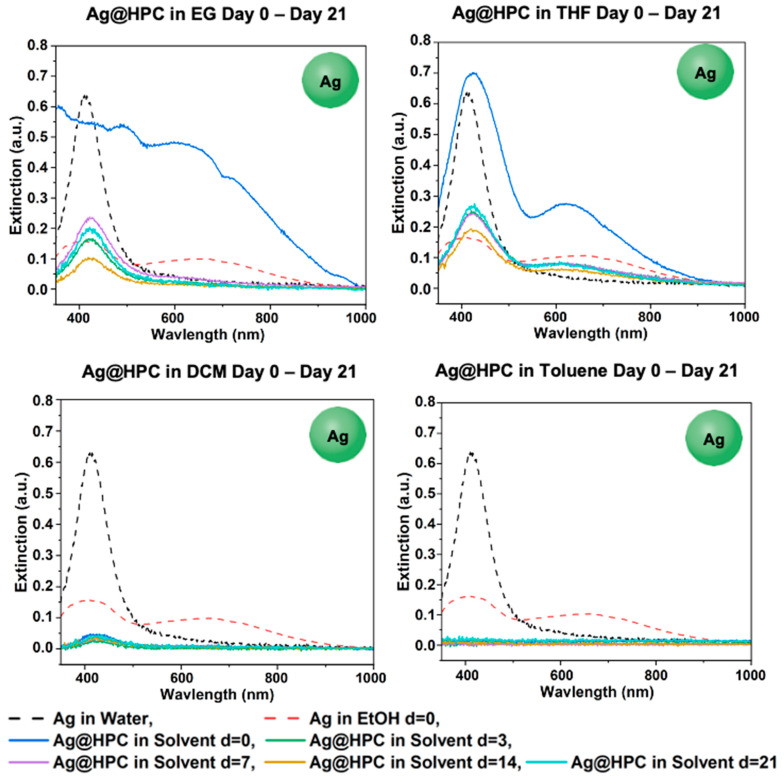
UV–vis spectra for Ag@HPC in EG, THF, DCM, and toluene after days 0, 3, 7, 14, and 21.

Ag@HPC survived in EtOH, EG, and THF from day 0 to day 21, as well as in DCM at low concentrations (Figure 10). However, Ag@HPC aggregated in toluene immediately following phase-transfer on day 0. When comparing these results on day 21 for each solvent, Ag@HPC in THF had the highest concentration, followed by EG, EtOH, and DCM (Figure S16). This trend also closely followed the one seen by GS-NS@HPC, which survived the 21-day period in EG and THF (see Figure 4, Figure 5, Figure 9 and Figure S16). Both Ag@HPC and GS-NS@HPC survived in DCM, but the GS-NS@HPC in DCM concentration decreased on day 21 only, whereas the Ag@HPC concentration in DCM remained low throughout the 21-day period. Additionally, both Ag@HPC and GS-NS@HPC aggregated immediately following phase-transfer into toluene.

### 3.5. Comparative Analysis of the Final Ligand Stability for AgNPs

#### Final Stability of Ag@Ligand in EG, THF, DCM, and Toluene

**Figure 11:**
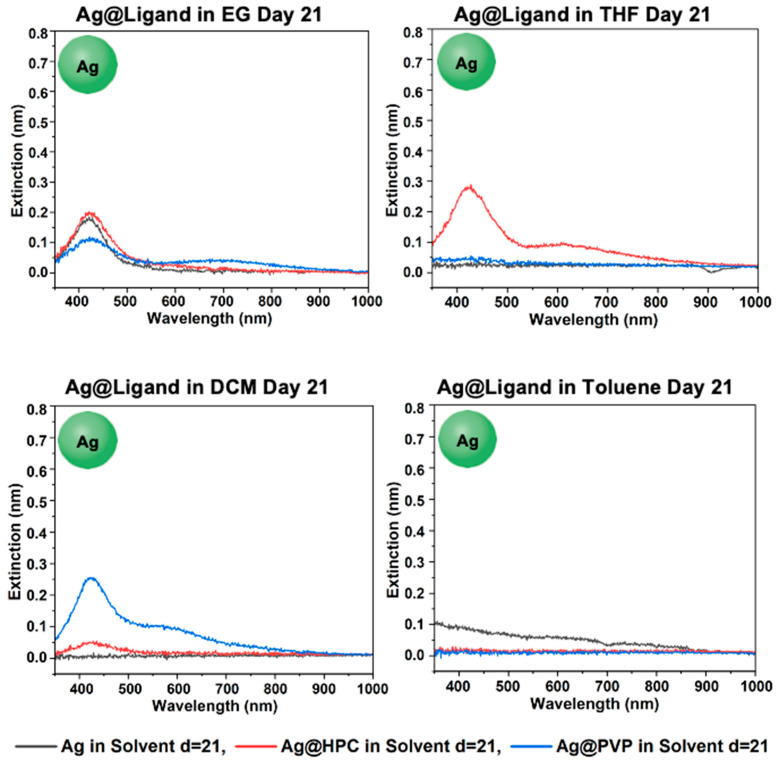
UV–vis spectra for Ag@Ligand in EG, THF, DCM, and toluene after day 21.

Final day 21 comparisons in the solvents in Figure 11 show that both Ag@HPC and Ag@PVP aggregated in toluene by day 21. While Ag@PVP survived phase-transfer into THF, the concentration of nanoparticles was very low. This also occurred with Ag@HPC in DCM. Due to the survival and high concentration of Ag@HPC in both EG and THF, and the partial survival of Ag@HPC in DCM, HPC was determined to have the highest effectiveness of phase-transfer among the ligands tested. This is similar to GS-NS@HPC, which effectively survived phase-transfer into all solvents except toluene (see Figure 7 and Figure 11). Overall, although, in the GS-NS structure formed by galvanic replacement, the Ag is known to be concentrated more on the inside of the nanoshell structure [66], stability of the AgNPs appears to track the GS-NSs closely in our studies with phase-transfer, likely confirming that the ligand effects are much more dominant in phase-transfer than nanoparticle composition.

### 3.6. Functionalization and Phase-Transfers of AuNPs

#### Ligand Exchange and Phase-Transfer Protocol for AuNPs

AuNPs with dimensions around 10–15 nm were prepared using chloroauric acid as the gold source and sodium citrate as the reducing agent, as reported previously [4]. The ligand functionalization for AuNPs was similar to GS-NSs and AgNPs; however, due to the smaller size of the AuNPs (~15 nm), the centrifugation and redispersion method was avoided, and direct shaking with the organic solvent was employed. Also, since the GS-NS and AuNP results showed aggregation with NaSt in most of the solvents, for the AuNPs study, only TX100, PVP, and HPC were used.

### 3.7. Comparative Analysis of the Final Ligand Stability for AuNPs

#### Phase-Transfer and Final Stability of Au@Ligand in EG, THF, DCM, and Toluene

**Figure 12:**
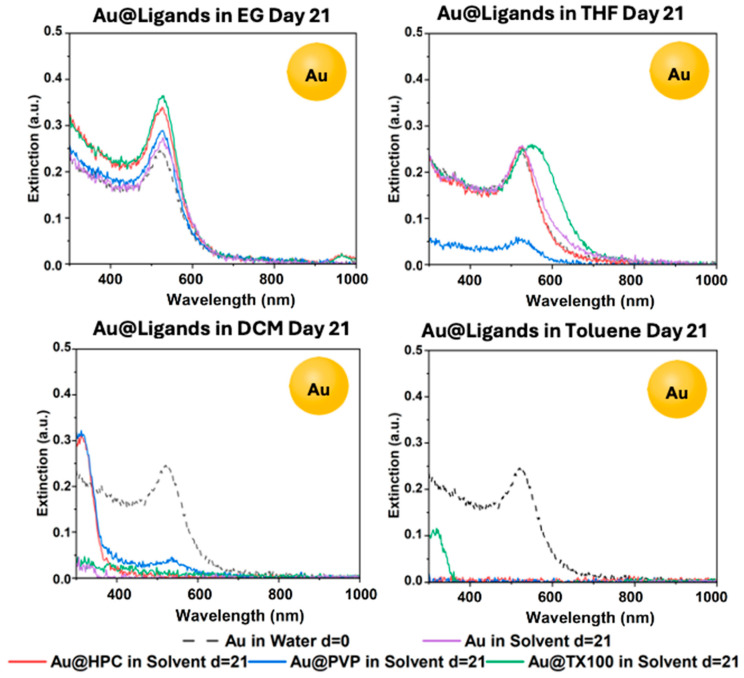
UV–vis spectra for Au@Ligand in EG, THF, DCM, and toluene after day 21.

The UV–vis spectra of the functionalized AuNPs after day 21 are shown in Figure 12, while Figures S17–S19 show the time-resolved data. As seen in Figure 12, after day 21, AuNPs treated with all tested ligands survived phase-transfer in EG. Nanoparticles treated with TX100 performed the best in EG. In THF, nanoparticles without ligands and treated with HPC and TX100 phase-transferred similarly, while the sample treated with PVP mostly aggregated. The only sample that achieved minimal phase-transfer in DCM was Au@PVP, while the rest did not show an LSPR signal in the DCM phase. This is slightly different from our observations with GS-NSs and AgNPs, where HPC-functionalized particles were stable in DCM. This difference arises due to the difference in methodology in the AuNPs study, where we did not centrifuge the particles before phase-transfer but simply shook them with the new solvent. Furthermore, without the ethanol-mediated method, the ligand stabilizers can only take effect at the interface between the two immiscible solvents, limited by poor interfacial contact, leading to a failure to transfer. This has been documented in previous studies as well [35]. Therefore, this spectrum is not an indication of aggregation in DCM but simply a lack of preference to move into the DCM layer when an aqueous phase is also present.

As seen in the time-resolved spectra in Figure S17, Au@TX100 survived phase-transfer in EtOH, EG, and THF throughout all 21 days. A red shift is observed in THF, which indicates some aggregation-induced size increase, although the colloids remain stable for all the days. In both the immiscible solvents DCM and toluene, the nanoparticles immediately aggregated or did not transfer. As seen in Figure S18, Au@PVP completely survived phase-transfer in EtOH and EG throughout the 21 days but mostly aggregated in THF after day 0 and maintained a very low concentration until day 21. In DCM, Au@PVP completely aggregated after day 3. Toluene had no observable phase-transfer regardless of the ligand on any of the days. Thus, similar to GS-NS@HPC and Ag@HPC, Au@HPC (Figure S19) survived phase-transfer into EG and THF throughout all 21 days and maintained a very low colloidal concentration in DCM.

## 4. Conclusions

A detailed investigation into the phase-transfer of citrate-stabilized GS-NSs into organic solvents with environmentally safe ligands was conducted. By studying these interactions, a couple of conclusions can be made. To phase-transfer a nanoshell into organic solvents, a combination of interactions was typically needed to stabilize the colloids without change to morphology. The three major factors responsible for forming stable colloids were solvent viscosity, dipole–dipole interactions, hydrogen bonding, and steric size, which helped improve ligand–solvent interactions and/or particle–particle repulsive forces.

HPC was found to be the best ligand for phase-transfer due to HPC’s steric size and high number and concentration of polar hydroxyl groups capable of dipole–dipole interactions and hydrogen bonds. EG was the best solvent for forming stable colloids with all the ligands, since EG can not only form hydrogen bonds with ligands but also with itself, adding viscosity to the solution of nanoparticles for further stability to the colloids. DCM was able to form dipole attractions with ligands with polar groups such as HPC and PVP. THF showed reliable stability only with GS-NS@HPC due to the additional hydrogen-bonding possibilities. None of the GS-NSs were able to phase-transfer into toluene with stability, confirming that steric repulsions, while adding stability with HPC in THF and DCM, are ultimately not sufficient alone in the absence of ligand–solvent interactions.

On extending the study to AgNPs and AuNPs, the trends remained consistent, with EG able to disperse all the functionalized particles and HPC showing the broadest long-term stability across EG, THF, and DCM. This broad consistency in our trends across three different particles suggests that these results are expected to be reproducible in other citrate-based systems as well. Overall, this research significantly expands the understanding and utility of the industry standard citrate-stabilized metallic nanoparticles using non-toxic agents and opens the door for plasmonic applications in organic solvents such as EG, THF, and DCM. These findings may provide a foundational platform for future studies in plasmonic organic nanosensing, the integration of plasmonic nanoparticles with organic functional materials, and inkjet printing of metallic nanopatterns with tunable surface plasmon resonance signals.

## Figures and Tables

**Table 1:** GS-NSs tabulated data of λ_max_, zeta potentials (ζ), hydrodynamic diameter (H.D.), and SEM particle size.

Particle	Property	Solvent					
		Water	EtOH	DCM	THF	EG	Toluene
**GS-NS**	Λ_max_ d = 21 (nm)	727	Agg	Agg	Agg	Agg	Agg
	ζ (mV)	−42	N/A	N/A	N/A	N/A	-
	H.D. (nm)	74.1	N/A	N/A	N/A	N/A	N/A
	Particle Size (nm)	46.5	N/A	N/A	N/A	N/A	N/A
**GS-NS@HPC**	Λ_max_ d = 21 (nm)	663	666	790	753	749	Agg
	ζ (mV)	−28.5	−21.7	-	-	-	-
	H.D. (nm)	96.2	125.9	221.7	154.9	129.4	N/A
	Particle Size (nm)	42.9	50.7	50.7	51.2	43.5	N/A
**GS-NS@PVP**	Λ_max_ d = 21 (nm)	728	752	788	Agg	750	Agg
	ζ (mV)	−42.8	−1.21	-	N/A	-	-
	H.D. (nm)	76.1	144.9	306.1	N/A	29.6	N/A
	Particle Size (nm)	44.1	42.1	50.6	N/A	49.1	N/A
**GS-NS@NaSt**	Λ_max_ d = 21 (nm)	728	Agg	Agg	Agg	803	Agg
	ζ (mV)	−51.4	N/A	N/A	N/A	-	-
	H.D. (nm)	87.1	N/A	N/A	N/A	Agg	N/A
	Particle Size (nm)	48.7	N/A	N/A	N/A	N/A	N/A
**GS-NS@TX100**	Λ_max_ d = 21 (nm)	742	Agg	Agg	663	761	782
	ζ (mV)	−31.3	N/A	N/A	-	-	-
	H.D. (nm)	76.9	N/A	N/A	Agg	97.7	684.8
	Particle Size (nm)	47.9	N/A	N/A	Agg	59.9	73.2

where: Agg = UV-vis spectra collected, but λ_max_ cannot be determined since aggregation caused signal flatlining; N/A = measurement not applicable due to aggregation; “-” = measurement not conducted due to instrument-solvent incompatibility.

## Data Availability

The original contributions presented in this study are included in the article/Supplemental Materials. Further inquiries can be directed to the corresponding author.

## Supplementary Materials

The following supporting information can be downloaded at: https://www.mdpi.com/article/10.3390/ma18225246/s1. Figure S1: UV-vis spectra for AgNPs individual batches. The LSPRs range from 405 to 410 nm in wavelength. Figure S2: UV-vis spectra for AgNPs master batch. The large AgNPs batch plotted with a peak localized surface plasmon resonance at 405 nm. Figure S3: UV-vis spectra for GS-NSs individual batches. The nanoparticles are within the expected 700–800 nm LSPR range. Figure S4: UV-vis spectra for GS-NSs master, giving an LSPR peak at 750 nm. Figure S5: SEM image of GS-NS at d = 21 in water after synthesis. The monodispersed nanoshells retained the spherical shape from the AgNP core. Figure S6: SEM images of (a) GS-NS@HPC (b) GS-NS@PVP (c) GS-NS@NaSt (d) GS-NS@TX100 all in water. Spherical nanoparticles are seen. Figure S7: UV-vis spectra for GS-NS@TX100 after days 0, 3, 7, 14, 21 in different solvents. Figure S8: SEM image of GS-NS@TX100 in toluene after day 21. Large aggregates and smaller colloids can be seen. The morphology of the nanoshells has changed from the original spherical shape. Figure S9: UV-vis spectra for GS-NS@NaSt after days 0, 3, 7, 14, 21 in different solvents. Figure S10: SEM images of GS-NS@PVP in EG and DCM on day 21. The nanoshells are still dispersed, and the shape of the particles has been maintained. Figure S11: TEM images of GS-NS@HPC on day 21 in EG and THF. No change in morphology is noticeable and a slight ligand corona can be seen in some nanoshells. Figure S12: (a) SEM of GS-NS@HPC, (b) TEM of GS-NS@HPC, (c) SEM of GS-NS@PVP and (d) SEM of GS-NS@TX100 after day 21 in EG. Particles maintained spherical shape and agglomerates are seen in GS-NS@HPC and GS-NS@TX100 after drying the nanoshells. Figure S13: (a) SEM of GS-NS@HPC phase-transferred to THF. Small spherical nanoshells are visible. (b) TEM of GS-NS@HPC phase-transferred to THF. Figure S14: SEM image of (a) GS-NS@HPC and (b) GS-NS@PVP phase-transferred to DCM after 21 days. Figure S15: SEM image of GS-NS@TX100 phase transferred to toluene after 21 days. Large aggregates are visible. Figure S16: UV-vis spectra for Ag@PVP and Ag@HPC after day 21 in Water, EtOH, EG, THF, DCM, and Toluene. Figure S17: UV-vis spectra for Au@TX100 in EG, THF, DCM, and toluene after days 0, 3, 7, 14, 21. Figure S18: UV-vis spectra for Au@PVP in EG, THF, DCM, and toluene after days 0, 3, 7, 14, 21. Figure S19: UV-vis spectra for Au@HPC in EG, THF, DCM, and toluene after days 0, 3, 7, 14, 21.
