# Supplementary material for: Stabilizing the Localized Surface Plasmon Resonance (LSPR) of Citrate-Synthesized Metal Nanoparticles in Organic Solvents
Source: Materials (Basel). 2025 Nov 20;18(22):5246. doi: 10.3390/ma18225246 (PMC12654536; doi:10.3390/ma18225246)
Supplement: Supplementary file 1 [file materials-18-05246-s001.zip › materials-3950684-supplementary.pdf]

materials

MDPI

Supporting Information

for

Stabilizing the Localized Surface Plasmon Resonance (LSPR) of Citrate-Synthesized Metal Nanoparticles in Organic Solvents

Jacob P. Magdon [1], Matthew J. Jasienski [1], Madison R. Waltz [1], Gabrielle A. Grzymski [1], Calvin Chen [1], Arion M. Solomon [1], Minh Dang Nguyen [2], Jong Moon Lee [2], John C. Deàk [1], T. Randall Lee [2] and Riddhiman Medhi [1,*]

[1] Department of Chemistry, University of Scranton, 800 Linden Street, Scranton, PA 18510, USA;
[2] Department of Chemistry and the Texas Center for Superconductivity, University of Houston, 4800 Calhoun Road, Houston, TX 77204-5003, USA

S1. Experimental Methods

S1.1. Materials

All materials were used without any additional modifications from the suppliers unless otherwise stated. Hydroxypropyl cellulose (HPC), MW ~100,000 was purchased from Sigma-Aldrich, polyvinylpyrrolidone (PVP), MW ~1,300,000 was purchased from Sigma-Aldrich, Triton X-100 from Sigma-Aldrich, L-ascorbic acid (99.6%) was purchased from Fisher Scientific, silver nitrate (99.99%) was purchased from Sigma-Aldrich, sodium citrate dihydrate was purchased from Fisher Scientific, potassium iodide was purchased from Fisher Scientific, potassium carbonate was purchased from Fisher Scientific, hydrogen tetrachlorauraute (III) hydrate (99.8%) was purchased from Stream, ethyl alcohol 100% was purchased from Pharmco, ethylene glycol (EG) was purchased from Fisher Scientific, tetrahydrofuran (THF) was purchased from Sigma-Aldrich, toluene was purchased from Sigma-Aldrich, dichloromethane (DCM) was purchased from Sigma-Aldrich, sodium stearate (NaSt) was made from stearic acid work up with sodium hydroxide purchased from Fisher Scientific and then recrystallized.

S1.2. Particle Synthesis

Silver nanoparticles (AgNPs), gold-silver nanoshells (GS-NSs), and gold nanoparticles (AuNPs) were synthesized according to previously reported methods.[1]

For Ag NPs, 0.0167 grams of silver nitrate was dissolved in 2.0 mL of water and 2.0 mL of 1% sodium citrate was added which released silver, nitrate, sodium, and citrate ions into solution. To this solution 50 uL of 7 uM potassium iodide was added and the solution was sonicated for 15 minutes. Meanwhile, a 24/40 250 mL round bottom flask was filled with 95.0 mL of water and brought up to 100 ^{0}C with stirring at 350 RPM in an oil bath. After 100 ^{0}C was reached, 1.0 mL of 5 mM ascorbic acid was added to the boiling water and the temperature was increased to 120^0 C. At 120 ^{0}C the solution of silver ions and potassium iodide was added to the boiling water. This reaction was refluxed for 1 hour, then allowed to cool to room temperature. The particles are then centrifuged for 15 minutes at 8000 RPMs to collect the precipitate AgNPs. The top solution was decanted out, and the particles were combined and redispersed in fresh Milli-Q water to 12.5 mL of

total volume. Multiple batches of AgNPs synthesized using this method were then combined to form a master batch for subsequent steps of functionalization and phase-transfer, to normalize any variation from the individual AgNPs batches. The AgNPs are stored in a fridge and in high concentration are greenish-grey in color. This method is used to make quasi-spherical AgNPs consistently. The size of the final silver nanoparticles was characterized by UV-vis spectroscopy and DLS.

For GS-NS, a solution of K-gold was made to perform galvanic replacement on the silver nanoparticles and form the gold-silver nanoshell.[1] A solution of K-gold was made by stirring 0.1000 grams of potassium carbonate into 400 mL of water with a stir bar at 450 RPMs for 5 minutes. After 1.5 mL of a 1% solution of gold chlorate was added. The solution turned yellow to clear after about 30 minutes of stirring at 450 RPMs. The K-gold was covered in aluminum foil and stored in a fridge overnight to be used the next day. Subsequently, 400 mL of the K-gold solution is distributed into four 100 mL Erlenmeyer flasks. 10.0 mL of silver nanoparticles was added to the 100 mL of the clear K-gold solution. This solution was stirred for 5 hours at 450 RPMs. The GS-NSs were then centrifuged for 15 minutes at 8000 RPMs, combined, and diluted to 10.0 mL with water. These particles are stored at room temperature. A diagram of this synthesis can be seen in Scheme 1.

For Au NPs, in a 500-mL beaker, a solution containing 1% $HAuCl_4$ (2 mL) was diluted with H_2O (to 400 mL) and then heated to boiling with stirring. An aliquot of a 1.0 wt% sodium citrate solution (10 mL) was added quickly to the hot $HAuCl_4$ solution and boiled for about 25 min until the color changed from light yellow to dark red.

S1.3. Characterization Methods

UV-visible spectroscopy was performed on a Thermo Fisher Scientific Evolution 300 by dilution of GS-NS samples at 5x and AgNP samples at 60x dilution. Five separate UV-vis spectrums were taken of a sample: once at the time of the phase transfer, at three days, at one week, two weeks, and three weeks. Dynamic light scattering was performed in a similar process by 5x dilution of GS-NS samples and 60x dilution of AgNPs. Samples were taken at 0 days and 21 days after synthesis to monitor the change of the hydrodynamic diameter. A scanning electron microscope (SEM, LEO-1525) with an accelerating voltage of 15 kV was used to image the nanoparticles. All SEM samples were deposited on a silicon wafer, cleaned with water and ethanol. The nanoparticles were also evaluated using a JEOL JEM-2010 transmission electron microscope (TEM), JEM-2000 FX TEM and JEM-2100 FX TEM; operating at an accelerating voltage of 200 kV. All TEM samples were deposited on 300 mesh holey carbon-coated copper grids and dried overnight before analysis. DLS and zeta potentials were performed using the Malvern Zetasizer Nano ZS instrument, SEM was taken of each sample that was unaggregated at the end of 21 days.

S2. Results and Discussion

S2.1. Synthesis and Morphology

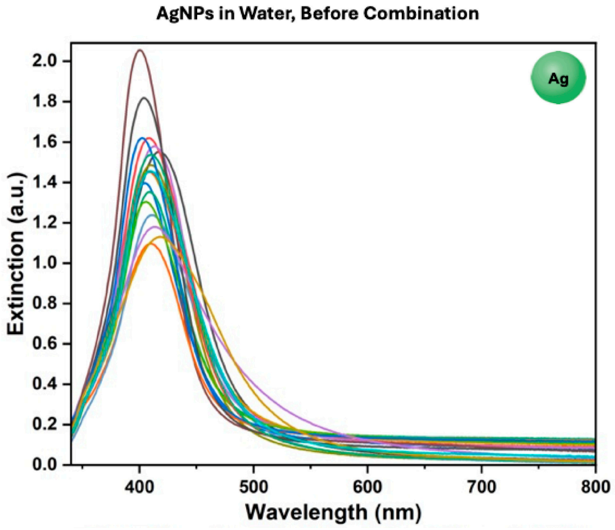

Figure S1. UV-vis spectra for AgNPs individual batches. The LSPRs range from 405 to 410 nm in wavelength.

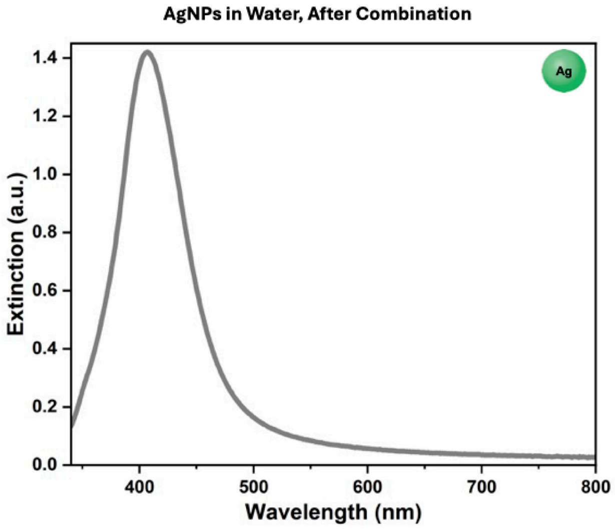

Figure S2. UV-vis spectra for AgNPs master batch. The large AgNPs batch plotted with a peak localized surface plasmon resonance at 405 nm.

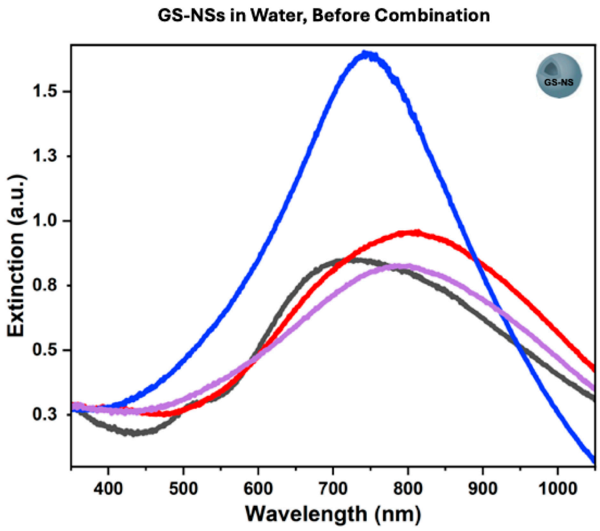

Figure S3. UV-vis spectra for GS-NSs individual batches. The nanoparticles are within the expected 700 – 800 nm LSPR range.

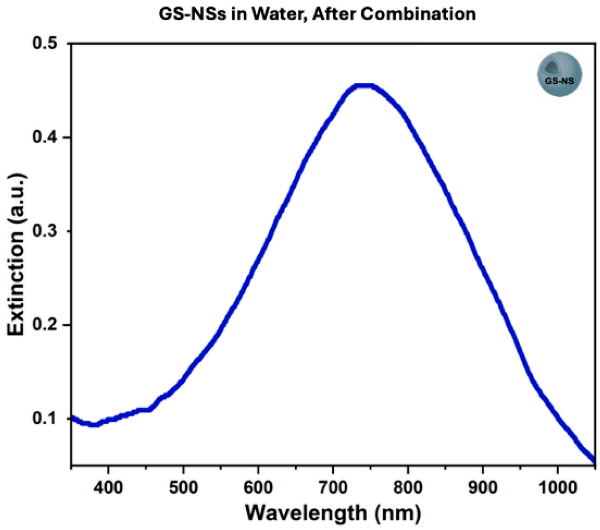

Figure S4. UV-vis spectra for GS-NSs master batch, giving an LSPR peak at 750 nm.

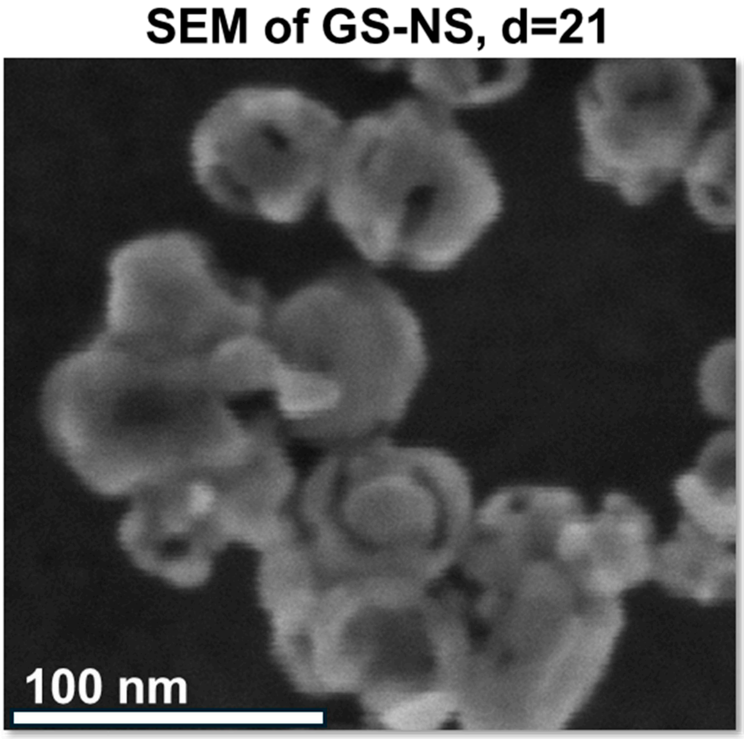

Figure S5. SEM image of GS-NS at d=21 in water after synthesis. The monodispersed nanoshells retained the spherical shape from the AgNP core.

S2.2. Functionalization and Phase Transfers of GS-NS

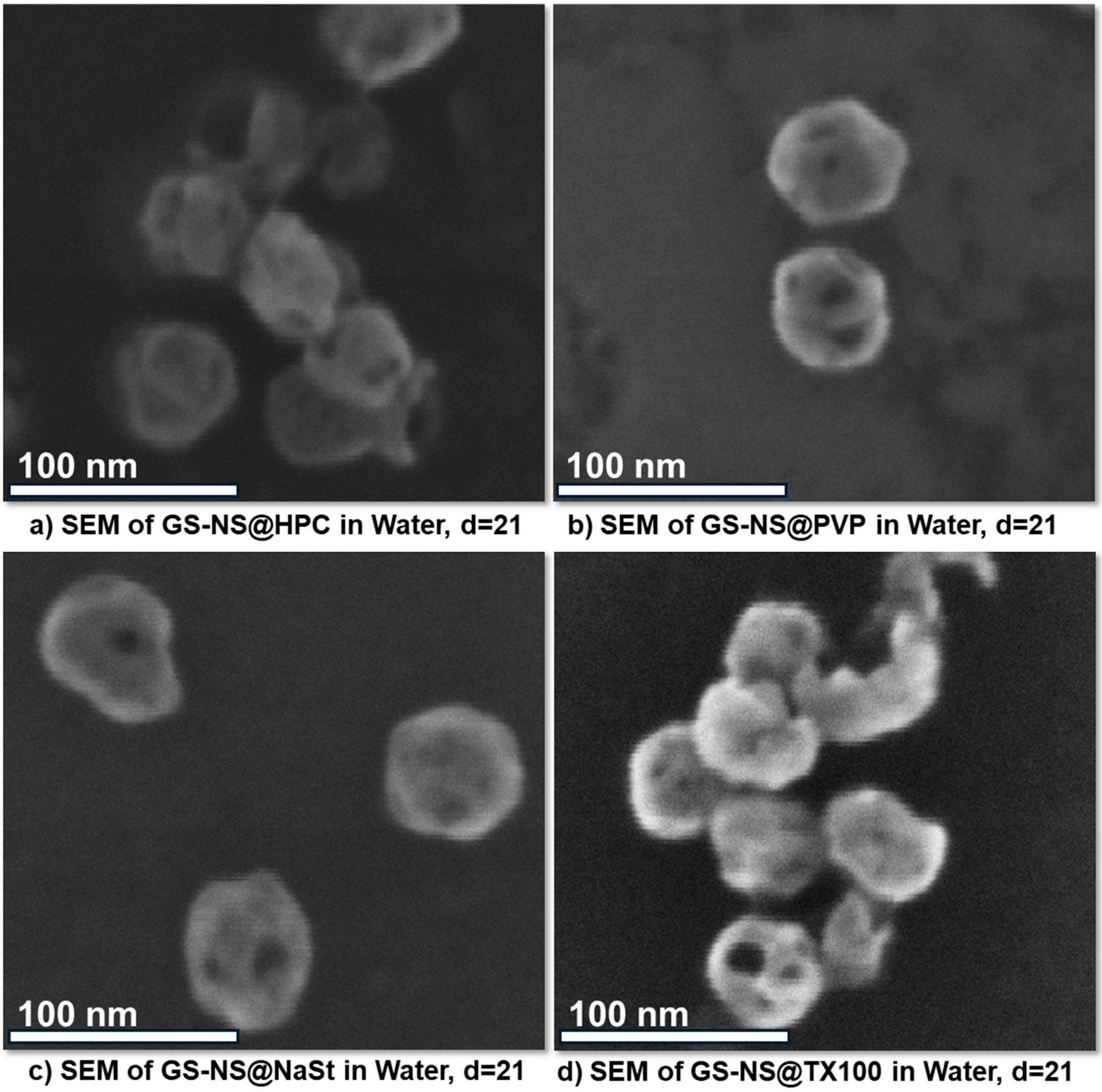

Figure S6. SEM images of a) GS-NS@HPC b) GS-NS@PVP c) GS-NS@NaSt d) GS-NS@TX100 all in water. Spherical nanoparticles are seen.

S2.2.1. Phase transfer of GS-NS@TritonX-100

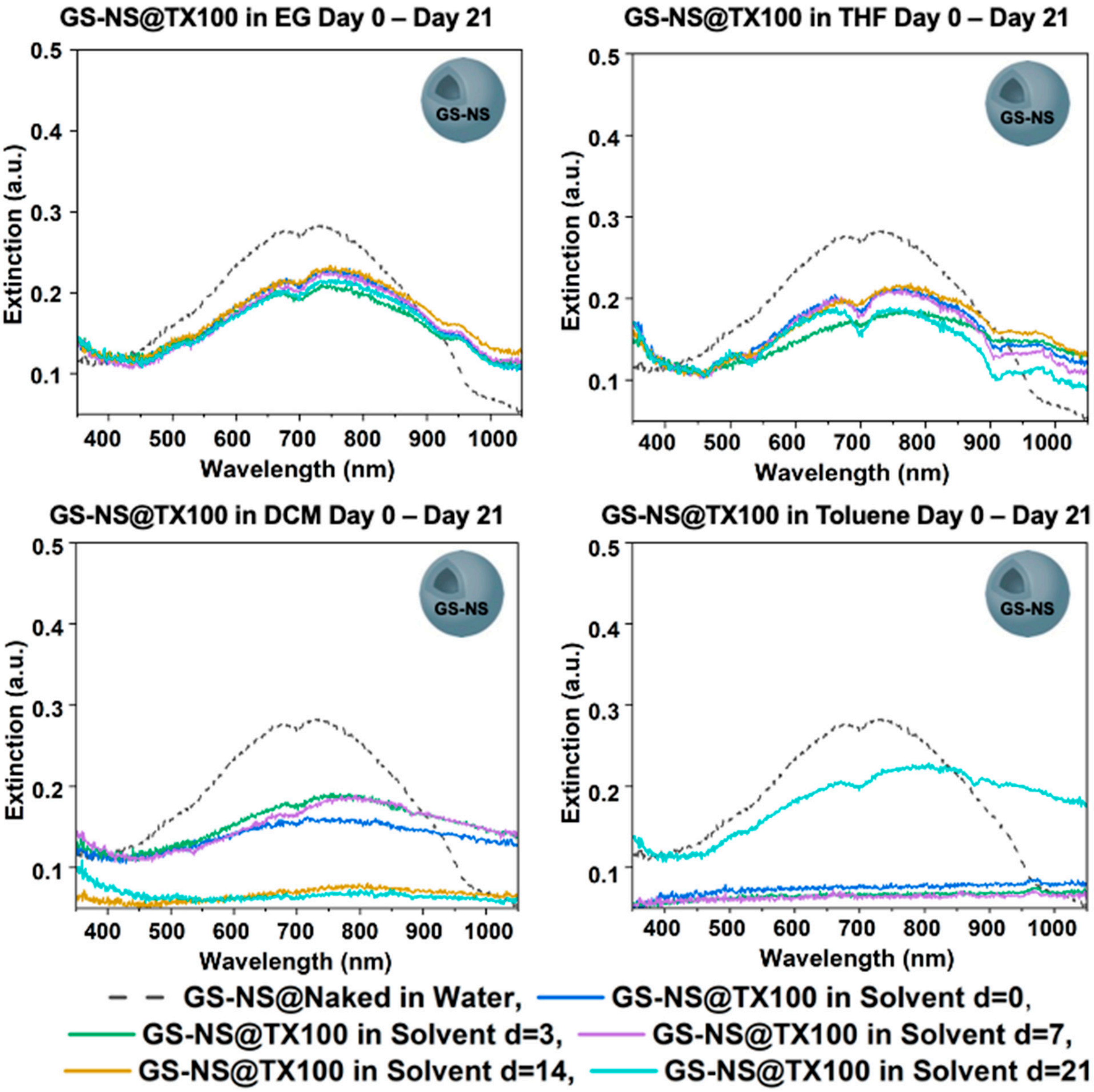

Figure S7. UV-vis spectra for GS-NS@TX100 after days 0,3,7,14,21 in different solvents.

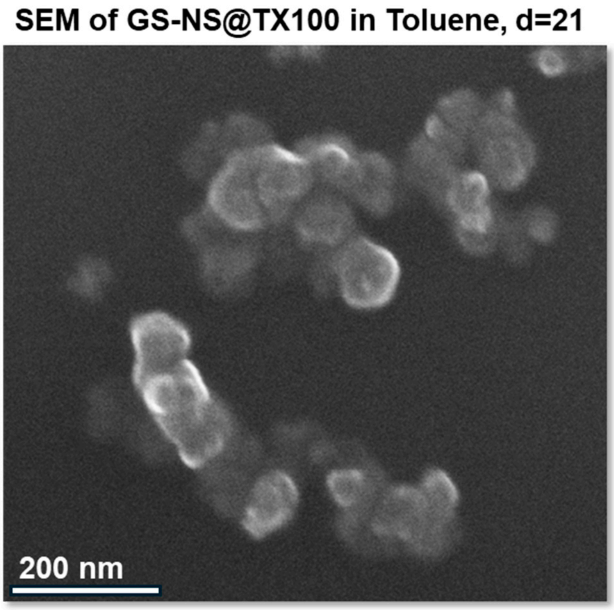

Figure S8. SEM image of GS-NS@TX100 in toluene after day 21. Large aggregates and smaller colloids can be seen. The morphology of the nanoshells has changed from the original spherical shape.

S2.2.2. Phase transfer of GS-NS@NaSt

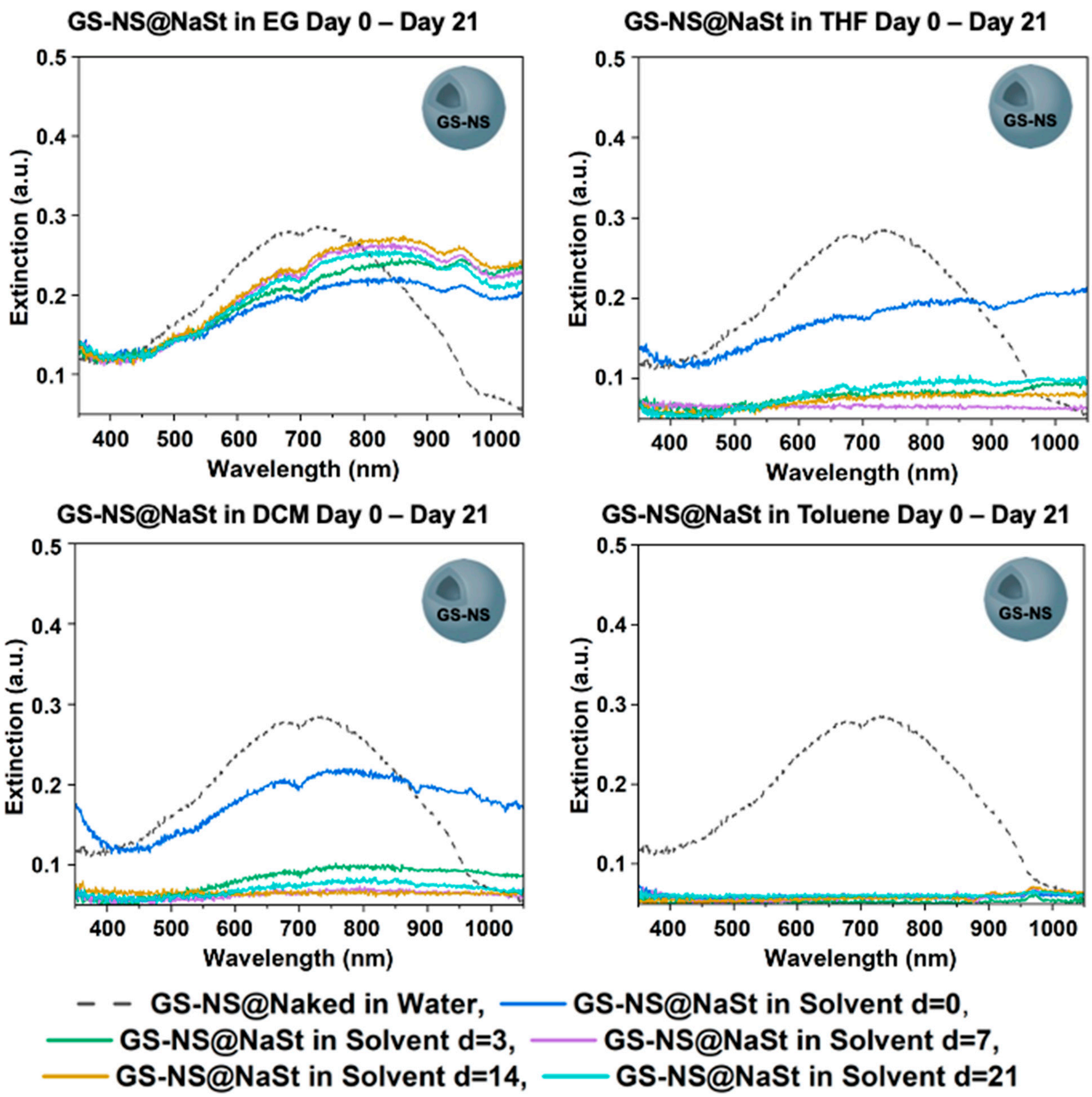

Figure S9. UV-vis spectra for GS-NS@NaSt after days 0,3,7,14,21 in different solvents.

S2.2.3. Phase transfer of GS-NS@PVP

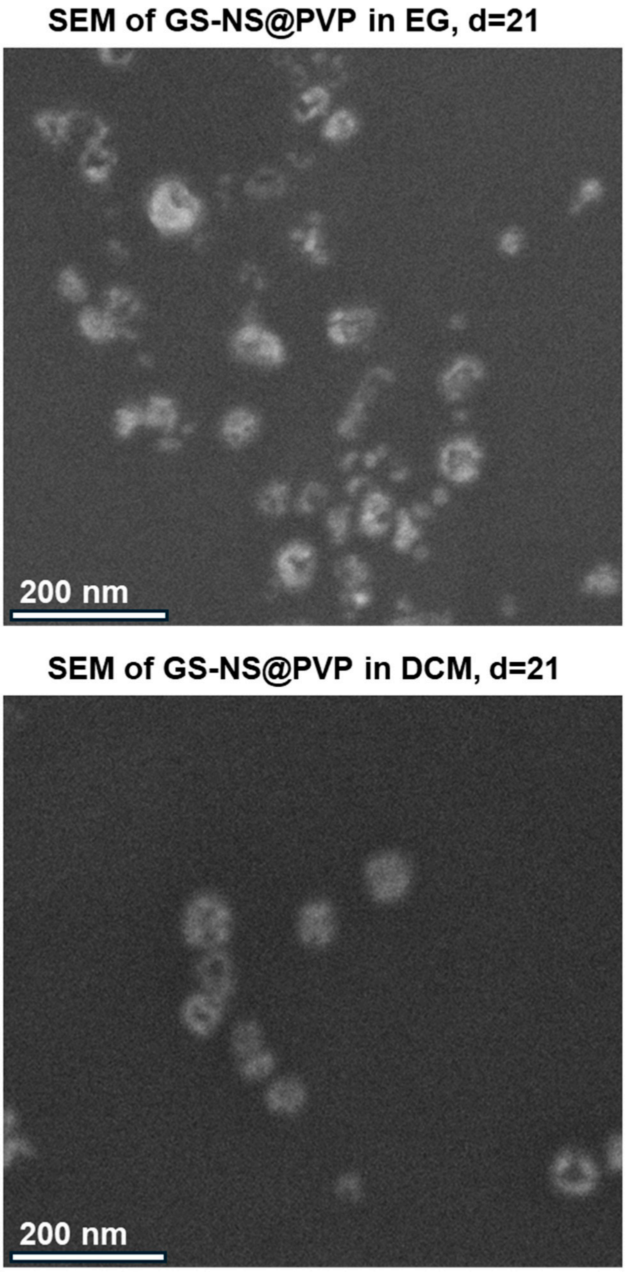

Figure S10. SEM images of GS-NS@PVP in EG and DCM on day 21. The nanoshells are still dispersed, and the shape of the particles has been maintained.

S2.2.4. Phase transfer of GS-NS@HPC

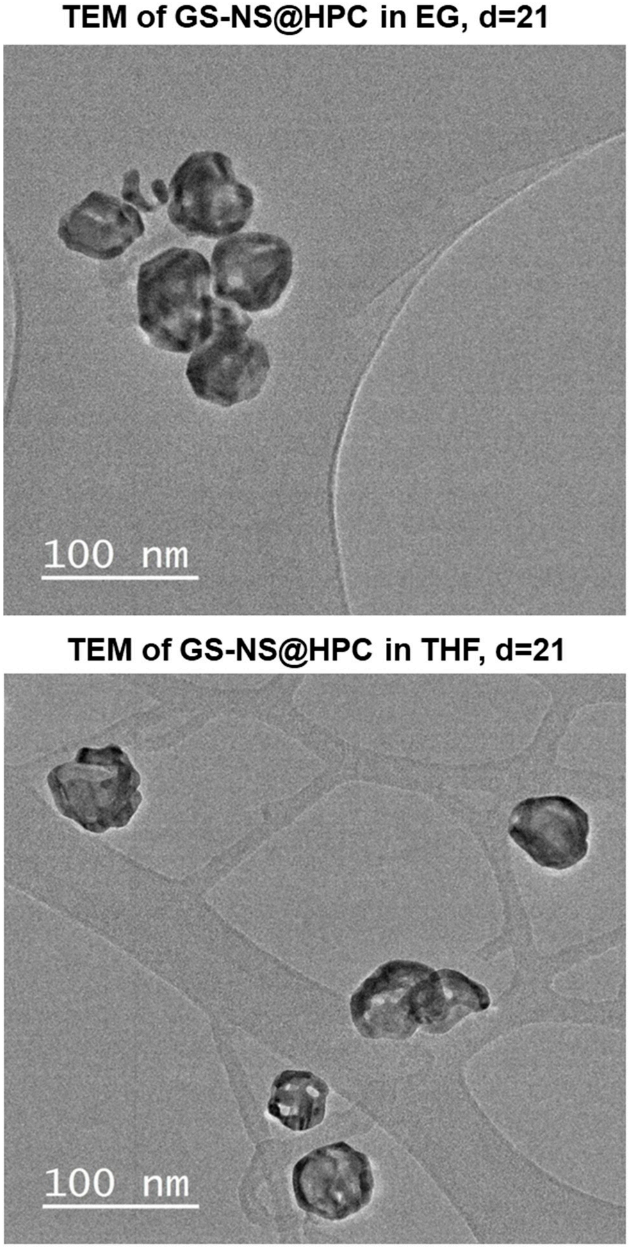

Figure S11. TEM images of GS-NS@HPC on day 21 in EG and THF. No change in morphology is noticeable and a slight ligand corona can be seen in some nanoshells.

S2.2.5 Comparative Analysis of the Ligands for Phase Transfer

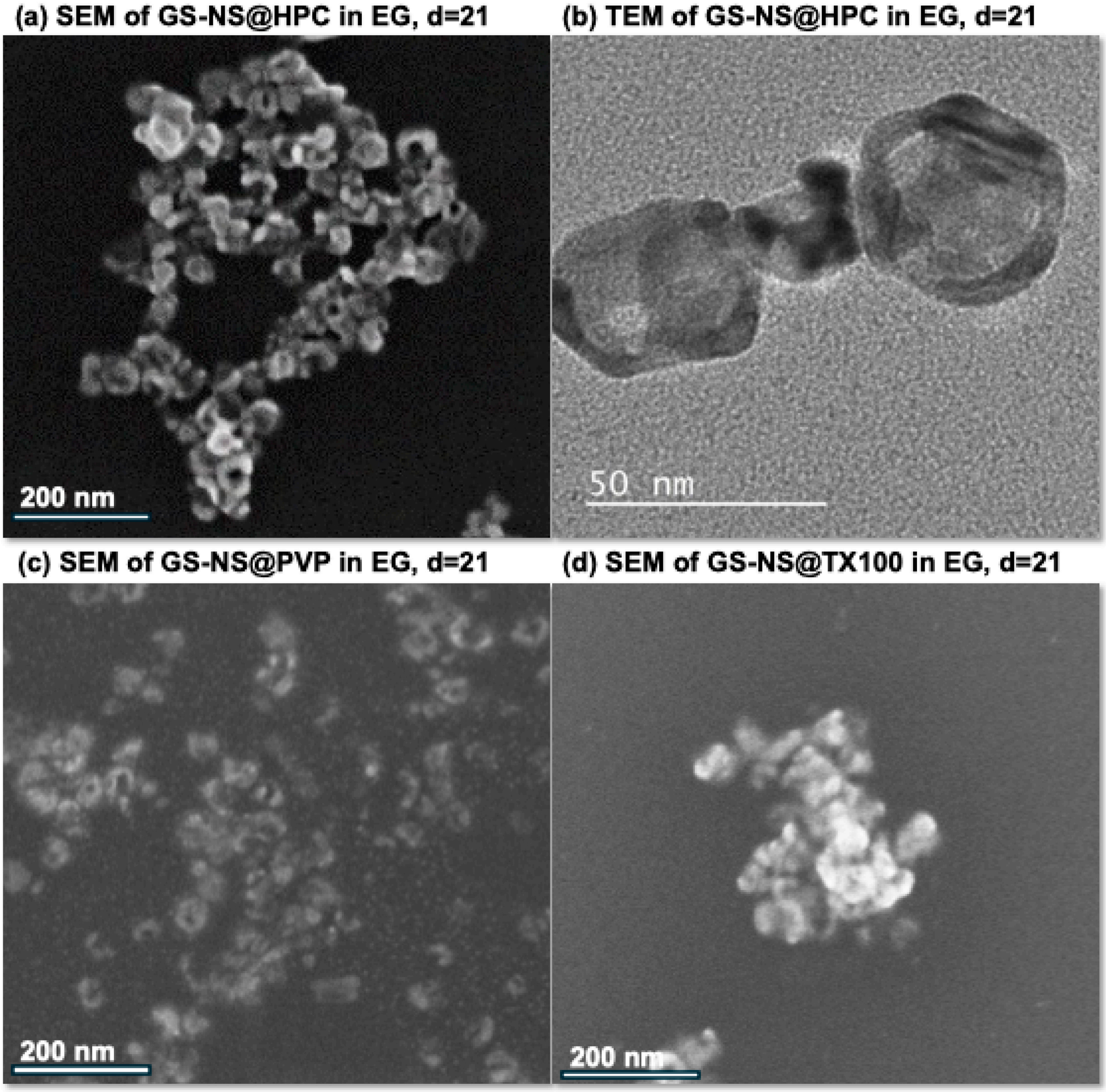

Figure S12. (a) SEM of GS-NS@HPC, (b) TEM of GS-NS@HPC, (c) SEM of GS-NS@PVP and (d) SEM of GS-NS@TX100 after day 21 in EG. Particles maintained spherical shape and agglomerates are seen in GS-NS@HPC and GS-NS@TX100 after drying the nanoshells.

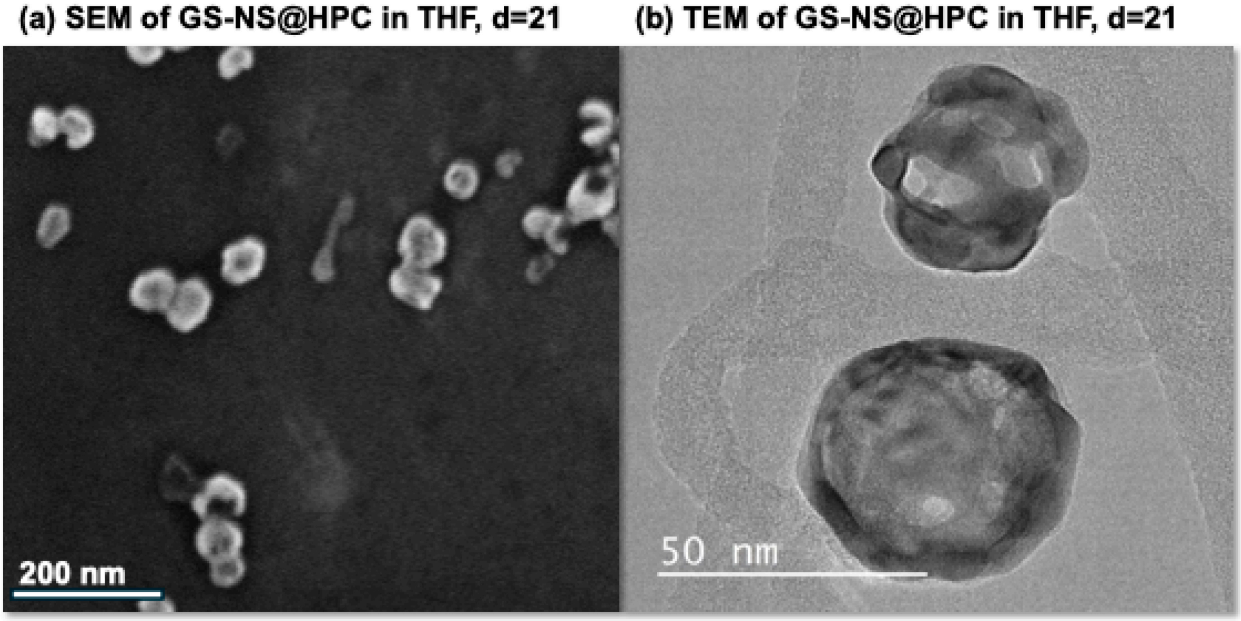

Figure S13. (a) SEM of GS-NS@HPC phase-transferred to THF. Small spherical nanoshells are visible. (b) TEM of GS-NS@HPC phase-transferred to THF.

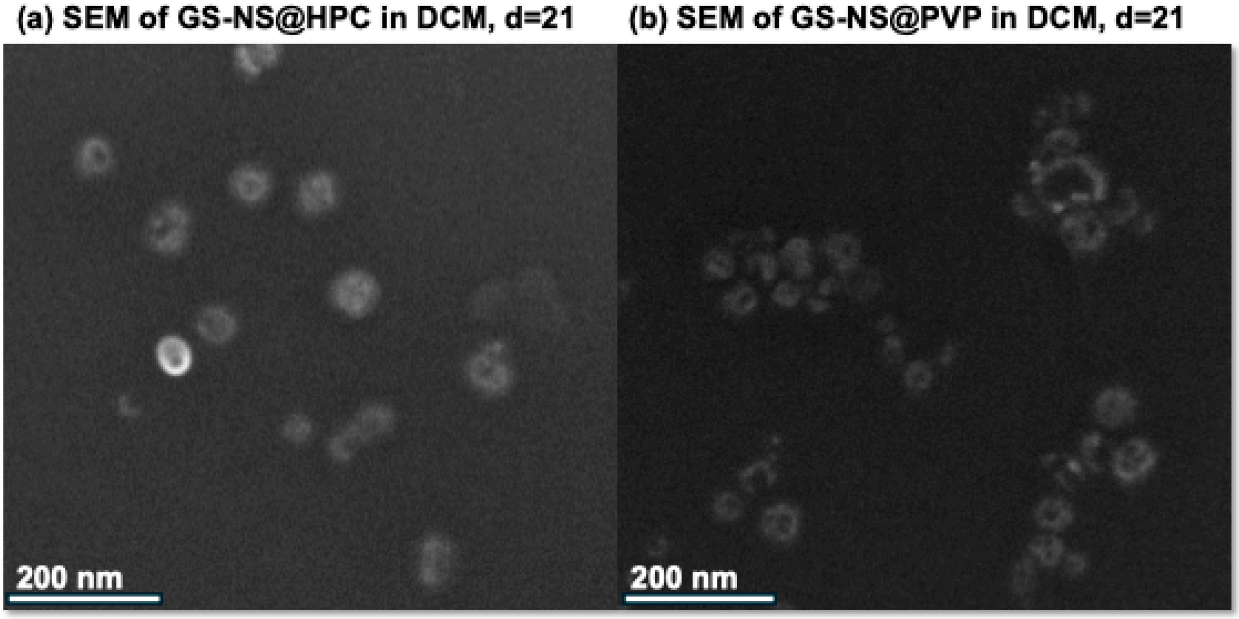

Figure S14. SEM image of (a) GS-NS@HPC and (b) GS-NS@PVP phase-transferred to DCM after 21 days.

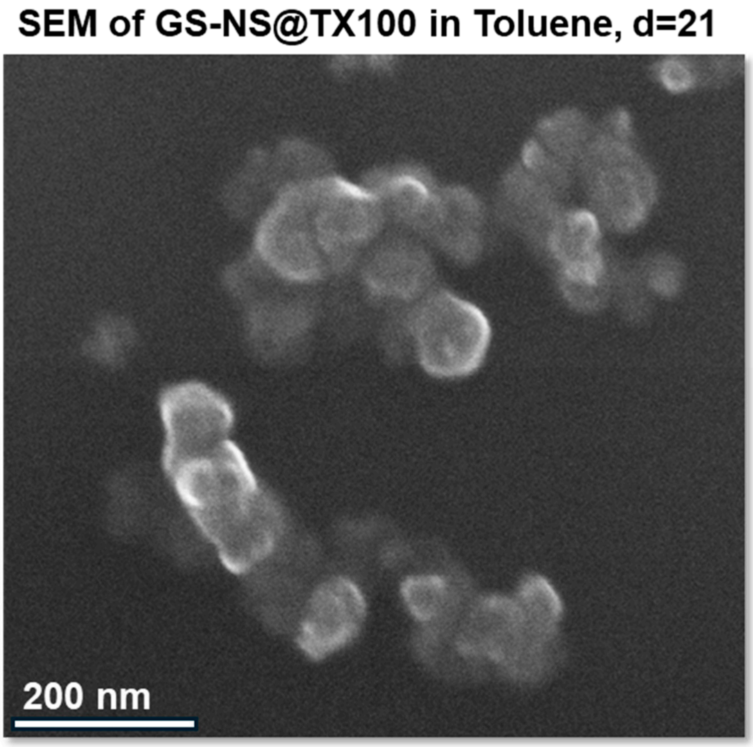

Figure S15. SEM image of GS-NS@TX100 phase-transferred to toluene after 21 days. Large aggregates are visible.

S2.3. Functionalization and Phase Transfers of AgNPs

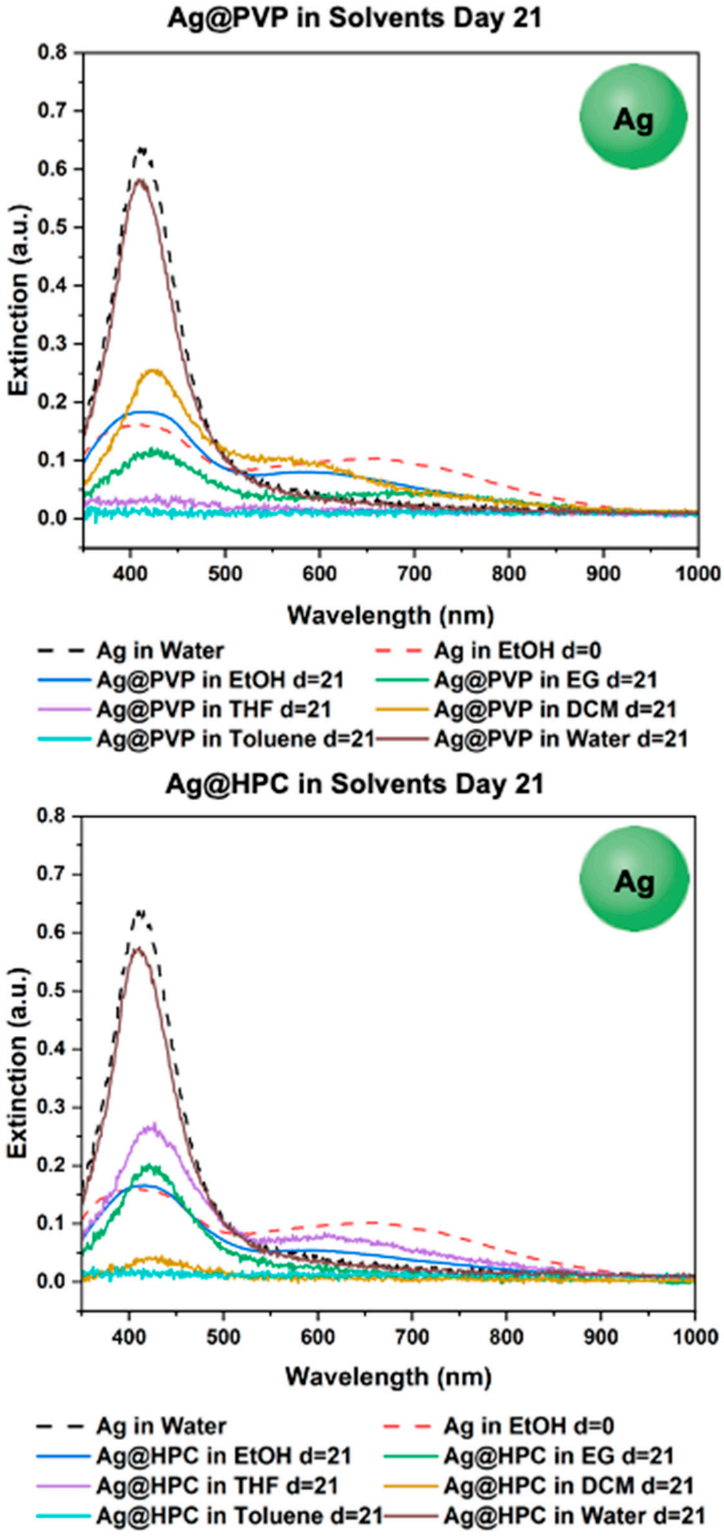

Figure S16. UV-vis spectra for Ag@PVP and Ag@HPC after day 21 in Water, EtOH, EG, THF, DCM, and Toluene.

S2.4. Functionalization and Phase Transfers of AuNPs

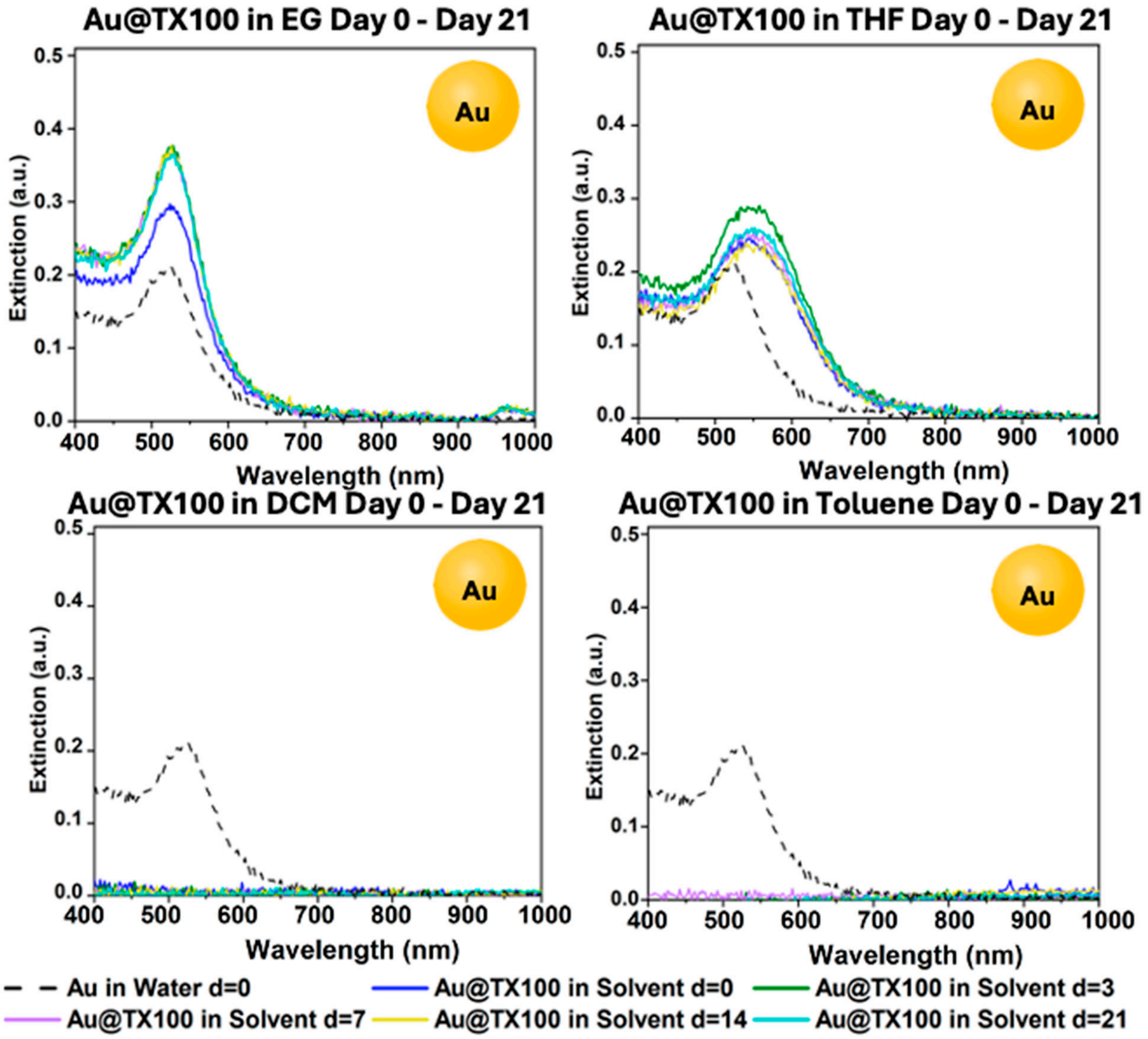

Figure S17. UV-vis spectra for Au@TX100 in EG, THF, DCM, and toluene after days 0,3,7,14,21.

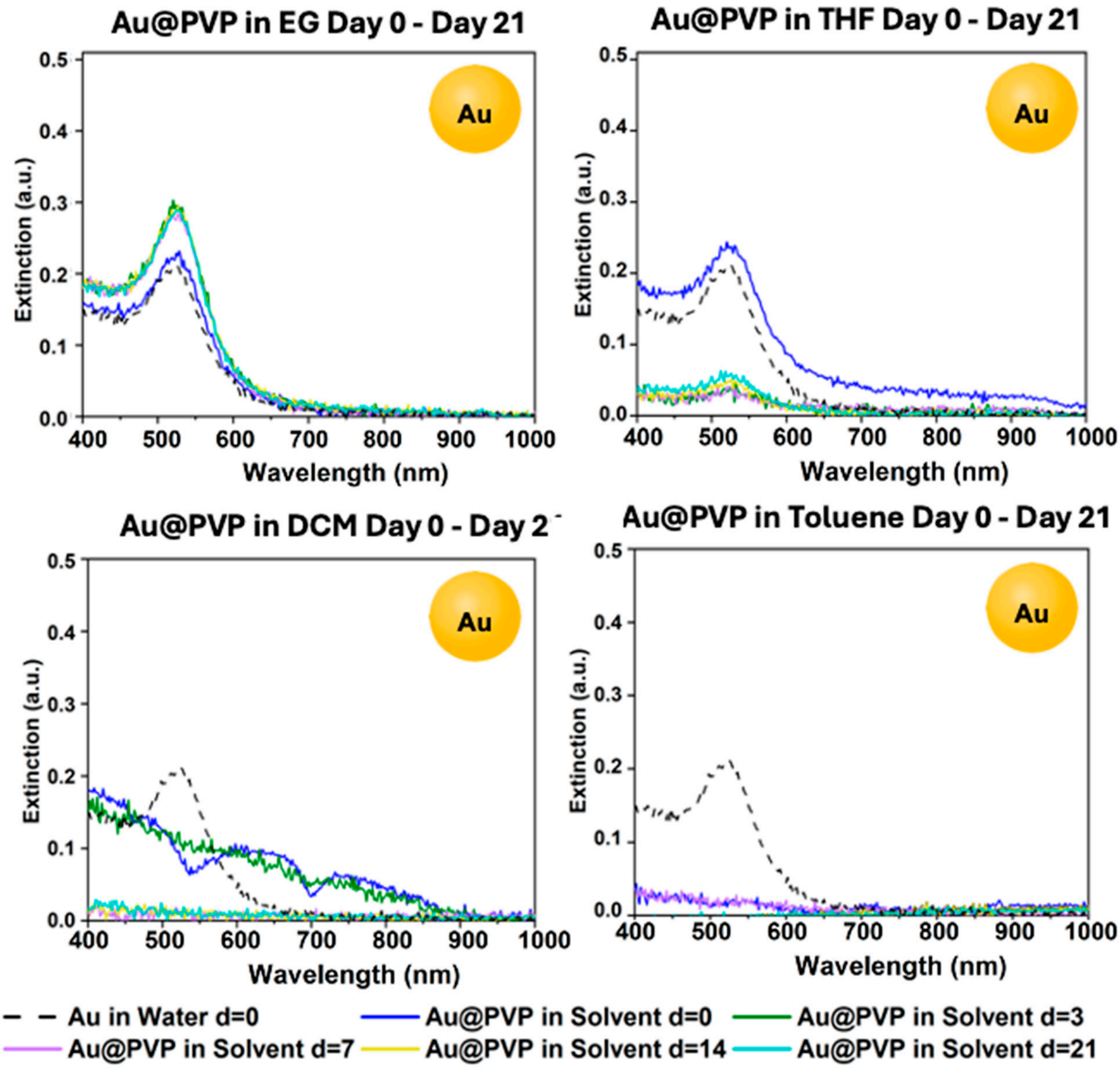

Figure S18. UV-vis spectra for Au@PVP in EG, THF, DCM, and toluene after days 0,3,7,14,21.

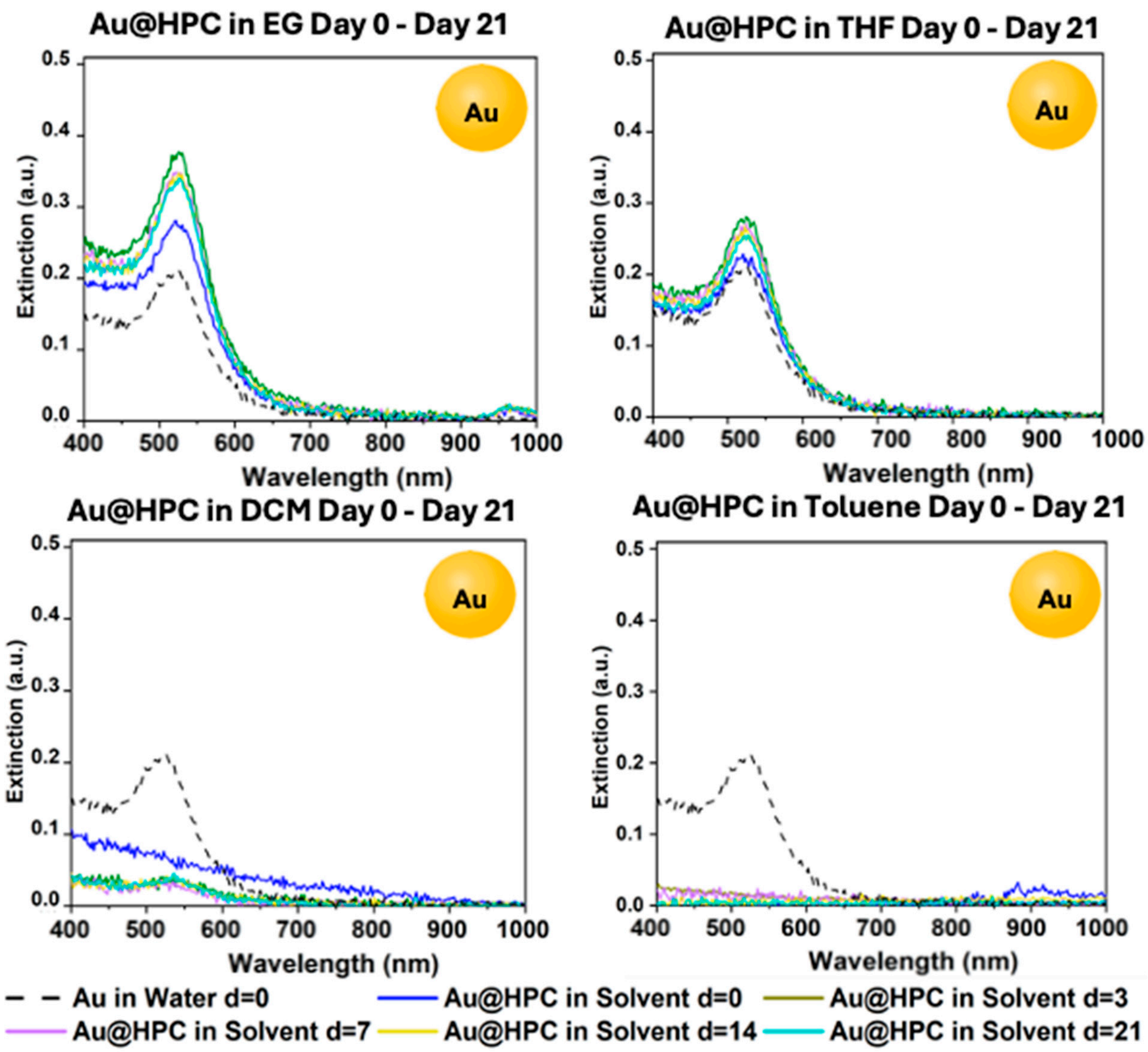

Figure S19. UV-vis spectra for Au@HPC in EG, THF, DCM, and toluene after days 0,3,7,14,21.

REFERENCES

(1) Medhi, R.; Li, C.-H.; Lee, S. H.; Srinoi, P.; Marquez, M. D.; Robles-Hernandez, F.; Jacobson, A. J.; Lee, T.-C.; Lee, T. R. Antimony- and Zinc-Doped Tin Oxide Shells Coated on Gold Nanoparticles and Gold–Silver Nanoshells Having Tunable Extinctions for Sensing and Photonic Applications. *ACS Appl. Nano Mater.* **2020**, *3*, 8958-8971.
